# Effect of competing mortality risks on predictive performance of the QFracture risk prediction tool for major osteoporotic fracture and hip fracture: external validation cohort study in a UK primary care population

**DOI:** 10.1136/bmjmed-2022-000316

**Published:** 2022-10-25

**Authors:** Shona J Livingstone, Daniel R Morales, Megan McMinn, Chima Eke, Peter Donnan, Bruce Guthrie

**Affiliations:** 1 Population Health and Genomics Division, University of Dundee, Dundee, UK; 2 Centre for Population Health Sciences, University of Edinburgh, Edinburgh, UK; 3 Advanced Care Research Centre, University of Edinburgh, Edinburgh, UK

**Keywords:** Primary health care, Rheumatology, Musculoskeletal diseases, Risk management, Preventive medicine

## Abstract

**Objective:**

To externally evaluate the QFracture risk prediction tool for predicting the risk of major osteoporotic fracture and hip fracture.

**Design:**

External validation cohort study.

**Setting:**

UK primary care population. Linked general practice (Clinical Practice Research Datalink (CPRD) Gold), mortality registration (Office of National Statistics), and hospital inpatient (Hospital Episode Statistics) data, from 1 January 2004 to 31 March 2016.

**Participants:**

2 747 409 women and 2 684 730 men, aged 30-99 years, with up-to-standard linked data that had passed CPRD checks for at least one year.

**Main outcome measures:**

Two outcomes were modelled based on the QFracture: major osteoporotic fracture and hip fracture. Major osteoporotic fracture was defined as any hip, distal forearm, proximal humerus, or vertebral crush fracture, from general practice, hospital discharge, and mortality data. The QFracture 10 year predicted risk of major osteoporotic fracture and hip fracture was calculated, and performance evaluated versus observed 10 year risk of fracture in the whole population, and in subgroups based on age and comorbidity. QFracture calibration was examined accounting for, and not accounting for, competing risk of mortality from causes other than the major osteoporotic fracture.

**Results:**

2 747 409 women with 95 598 major osteoporotic fractures and 36 400 hip fractures, and 2 684 730 men with 34 321 major osteoporotic fractures and 13 379 hip fractures were included in the analysis. The incidence of all fractures was higher than in the QFracture internal derivation. Competing risk of mortality was more common than fracture from middle age onwards. QFracture discrimination in the whole population was excellent or good for major osteoporotic fracture and hip fracture (Harrell’s C statistic in women 0.813 and 0.918, and 0.738 and 0.888 in men, respectively), but was poor to moderate in age subgroups (eg, Harrell’s C statistic in women and men aged 85-99 years was 0.576 and 0.624 for major osteoporotic fractures, and 0.601 and 0.637 for hip fractures, respectively). Without accounting for competing risks, QFracture systematically under-predicted the risk of fracture in all models, and more so for major osteoporotic fracture than for hip fracture, and more so in older people. Accounting for competing risks, QFracture still under-predicted the risk of fracture in the whole population, but over-prediction was considerable in older age groups and in people with high comorbidities at high risk of fracture.

**Conclusions:**

The QFracture risk prediction tool systematically under-predicted the risk of fracture (because of incomplete determination of fracture rates) and over-predicted the risk in older people and in those with more comorbidities (because of competing mortality). The use of QFracture in its current form needs to be reviewed, particularly in people at high risk of death from other causes.

**What is already known on this topic**

- The QFracture risk prediction tool is recommended by the National Institute for Health and Care Excellence (NICE) to predict the risk of fracture and to guide decisions to start bisphosphonates, on the basis of previous validation studies showing good predictive performance
- Previous validation studies have followed the QFracture derivation study in not including fractures recorded in hospital discharge data, and in not accounting for competing risk of mortality

**What this study adds**

- The observed incidence of fracture was higher in this study (which included hospital recorded fractures) than in the QFracture derivation and validation studies (which did not)
- Despite excellent discrimination in the whole population, systematic under-prediction of the risk of fracture by QFracture was found, as was systematic over-prediction in older people and in those with more comorbidities when accounting for competing risk of mortality
- The use of QFracture for clinical prediction needs reviewing, particularly in people at high risk of death from other causes

**How this study might affect research, practice, or policy**

- Clinicians should be aware that QFracture will under-estimate fracture risk in younger people, but over-estimate fracture risk in people at high risk of death from other causes
- Research is needed to examine the implications of competing mortality risk for other recommended clinical prediction tools where the time -horizon for prediction is long

## Introduction

Fragility or low impact fractures are a common consequence of osteoporosis and osteopenia, and a major cause of morbidity, disability and, in some cases, death. Bisphosphonates reduce the risk of hip and vertebral fractures in people with osteoporosis,^1^ and international guidelines recommend drug treatment for people at high risk of fracture.^1–5^ In the UK, guidelines recommend the use of a fracture risk prediction tool in middle aged and older people who have risk factors for fracture, with measurement of bone mineral density for further classification of risk in those at intermediate risk.^2 4^ In the US, guidelines from the US Bone Health and Osteoporosis Foundation (previously the National Osteoporosis Foundation) recommend similar use of prediction tools for middle aged people but also recommend routine measurement of bone mineral density in older people.^5^ These types of guideline recommendations based on risk are increasingly used by people who develop guidelines to target treatment to those with the greatest capacity to benefit, but the effectiveness of this strategy critically depends on the performance of the risk prediction tools used.

Many fracture risk prediction tools have been created, although only two have undergone repeated external validation: QFracture and Garvan.^6 7^ The first version of QFracture^8^ was externally validated in a UK primary care dataset, and was found to have excellent discrimination and calibration (discrimination is the ability of the prediction tool to correctly differentiate between people who have a fracture and those who have not, whereas calibration refers to how closely the predicted and observed probabilities agree).^9^ Subsequently, Dagan et al externally validated the updated QFracture algorithm and the Garvan prediction tool in an Israeli dataset. QFracture had good discrimination but Garvan had moderate discrimination, and both tools systematically under-predicted the risk of fracture.^7^

The fracture risk assessment tool (FRAX) has been internally validated in several datasets, with discrimination reported as good but calibration has rarely been assessed.^6 10^ FRAX cannot be externally validated, however, because the underlying FRAX algorithm has never been made public which prevents full independent evaluation.^7^ Dagan et al also presented an external validation of FRAX in their analysis, but FRAX predictions were not based on full FRAX estimates of risk because the prediction equation is not published.^7^ Based on the approximate FRAX risk used, considerable under-prediction of fractures for this tool was found.

In the UK, the National Institute for Health and Care Excellence (NICE) recommends the use of either QFracture or FRAX to inform decisions to start treatment with bisphosphonates, but recognises that the estimated risk of fracture for individuals can vary considerably between tools.^1 2^ FRAX over-predicted the risk of fracture when the same method of determining fractures as the QFracture derivation was used.^2 8 11^ Two possible reasons for these differences include how fractures are identified in the derivation of each tool, because QFracture uses codes in primary care records and mortality data^12^ and FRAX uses self-report and hospital records^13^ (these might be incomplete in different ways), and only FRAX takes into account competing risks of mortality. Competing risk of mortality from non-fracture causes is a known problem in risk prediction because standard modelling methods assume that patients who are censored before the intended end of follow-up have the same risk of fracture as those who are not censored. Although this assumption might be reasonable for loss to follow-up because of change in address, when someone dies the assumption is clearly false. Not accounting for competing risk of mortality over-predicts the risk of fracture, which is likely to be more of a problem in older people and those with multimorbidities.^14–16^ The aim of this study therefore was to externally validate the QFracture risk prediction tool, and specifically to compare prediction in relation to better determination of fracture rates, and to examine the effect of competing risk on predictive performance.

## Methods

### Data source and population

Linked general practice (Clinical Practice Research Datalink Gold), mortality registration (Office of National Statistics), and hospital inpatient (Hospital Episode Statistics) data were used. The data are similar to the QFracture derivation dataset in terms of inclusion of linked primary care and mortality data, but we also used linked hospital admission data to determine if a fracture occurred. To be included, patients had to be permanently registered with a general practice contributing up-to-standard (ie, passing Clinical Practice Research Datalink quality checks) data for at least one year; have linkage to Hospital Episode Statistics discharge data and Office of National Statistics mortality data; and aged ≥30 years and <100 years. Cohort entry was the latest of the dates on or after 1 January 2004. Cohort exit was the date of the earliest of the first relevant fracture event, death, deregistration from the general practice, date of the last data collection from the practice, or the end of the study on 31 March 2016. All outcomes and predictors were recorded blind to the study hypothesis and recorded as part of routine clinical care. No formal power calculation was done because the study size was determined by data available in the Clinical Practice Research Datalink, which was considered sufficient.^17^

### Outcomes

Two outcomes were modelled based on the QFracture tool: major osteoporotic fracture and hip fracture.^12^ Major osteoporotic fracture was defined as hip, vertebral, wrist, or proximal humeral fractures determined from codes in the general practice electronic health record (with Read codes, which have been shown to have high positive predictive value for hip fracture),^18^ Hospital Episode Statistics discharge diagnoses (ICD-10 (international classification of diseases, 10th revision) codes recorded in the primary position as the reason for admission to hospital), and Office of National Statistics death registration (ICD-10 codes). QFracture does not publish the codes used to define these outcomes, so we derived our own (online supplemental tables S1 and S2). Major osteoporotic fracture recorded before entry into the study was used as a predictor variable. Major osteoporotic fracture or hip fracture recorded after the index date was used as the outcome variable, with the date of the event taken as the first record of fracture.

10.1136/bmjmed-2022-000316.supp1 Supplementary data

### Prediction model

We used the published QFracture-2016 risk model (under GNU Lesser General Public Licence, version 3) and calculated the QFracture predicted 10 year risk of a major osteoporotic fracture and hip fracture for all patients in our cohort. Online supplemental tables S3-S5 describe the derived codelists for each morbidity predictor. The key difference from the QFracture derivation was that for QFracture, body mass index, alcohol consumption, and smoking status, recorded after the date of entry into the study but before any fracture outcome, could be used in the prediction, whereas in this analysis we restricted predictor values to those recorded before entry into the study only, to avoid the use of future information in the prediction.

### Comorbidity

For each patient at baseline, we calculated the Charlson comorbidity index based on primary care Read codes.^19^ The Charlson comorbidity index was not used in the prediction, but was used to classify the analysis of discrimination and calibration by level of comorbidity (Charlson comorbidity index score 0, 1, 2, and ≥3 groups).

### Missing data

Online supplemental table S6 details the extent and management of missing data. In common with the QFracture derivation, those with missing data for ethnic group were assumed to be white. For missing data on body mass index, smoking status, and alcohol consumption, multivariate imputation by chained equations^20^ was used to generate five imputed datasets, which were combined by using Rubin’s rules.^21^ Morbidities and prescribing used for prediction were assumed to be absent if there were no relevant data recorded for them (the same as for the QFracture derivation), reflecting that recording of morbidity and prescribing data in the Clinical Practice Research Datalink is generally good.^22 23^

### Statistical analysis

Based on the recommendations of reporting guidance,^24^ the initial analysis compared the study population and fracture rates in this study with the previously published QFracture derivation and validation cohorts (although variable reporting across previously published papers means that the comparison population varies depending on the data available).^8 9 12^ The performance of the QFracture-2016 risk score was assessed by examining discrimination and calibration. We used Harrell’s C statistic, shortened to only include pairs where the earliest survival time is no later than 10 years after entry (a C statistic of 0.5 indicates discrimination that is no better than chance, whereas a C statistic of 1 indicates perfect discrimination). Two other measures of discrimination were calculated, the D statistic of Royston and Sauerbrei (which is based on the separation in event free survival between patients with predicted risk scores above and below the median; higher values indicate greater discrimination),^25^ and a related R^2^ statistic estimating explained variation for censored survival data.^26^

Calibration was assessed for 10 equally sized groups (deciles) of participants ranked by predicted risk, by plotting observed proportions versus predicted probabilities. We estimated observed risk for censored data in two ways: with the standard Kaplan-Meier estimator (which is consistent with the assumptions made in the QFracture derivation in that it does not account for competing risks); and the Aalen Johansen estimator (an extension to allow for competing events, in this case, death from causes other than fractures).^27^ All models were fitted in R-4.0.0 and Stata 11.2. Plots were generated separately for sex, for all patients, and for subgroups for age and Charlson comorbidity index, based on summary statistics pooled across the imputed datasets.

### Patient and public involvement

Public contributors were involved in the design and conduct of the study as members of the study steering group.

## Results

We included 2 747 409 women and 2 684 730 men in the analysis, with mean ages of 50.7 and 48.5 years, respectively (table 1). The study population was similar to the previously published QFracture internal validation population in term of mean age, sex, body mass index, and ethnic group but we found a higher recorded prevalence of previous major osteoporotic fracture, residence in a nursing home or care home, and many long term conditions, including type 2 diabetes, history of falls, dementia, cancer, asthma or chronic obstructive pulmonary disease, chronic renal disease, malabsorption, and epilepsy or prescribed anticonvulsant drugs. For the population evaluated for major osteoporotic fracture, median follow-up was 5.7 (interquartile range 2.2-10.5) years in women and 5.6 (2.2-10.4) years in men. For hip fracture, median follow-up was 5.9 (2.2-10.6) years in women and 5.7 (2.2-10.4) years in men.

**Table 1:** Baseline data in our external validation cohort and in previously published QFracture internal validation cohort^12^

Characteristics	External validation cohort		QFracture internal validation cohort^12^*
	Women (n=2 747 409, 50.6%)	Men (n=2 684 730, 49.4%)	All patients (n=1 583 373)
Mean (SD) age (years)	50.7 (17.4)	48.5 (15.6)	50 (1.6)
Mean (SD) body mass index	26.6 (6.0)	27.1 (4.8)	26.1 (4.6)
Women	2 747 409 (50.6)		804 563 (50.8)
Ethnic group:			
White or not recorded	2 614 423 (95.2)	2 556 923 (95.2)	1 493 455 (94.3)
Indian	25 420 (0.9)	27 087 (1.0)	17 670 (1.1)
Pakistani	11 121 (0.4)	12 316 (0.5)	6489 (0.4)
Bangladeshi	3473 (0.1)	4972 (0.2)	4191 (0.3)
Other Asian	18 896 (0.7)	17 758 (0.7)	10 779 (0.7)
Black Caribbean	4780 (0.2)	4030 (0.2)	10 144 (0.6)
Black African	22 736 (0.8)	20 776 (0.8)	17 367 (1.1)
Chinese	7358 (0.3)	5517 (0.2)	5206 (0.3)
Other ethnic group	39 202 (1.4)	35 351 (1.3)	18 072 (1.1)
Smoking status:			
Non-smoker	1 146 025 (41.7)	807 294 (30.1)	773 198 (48.8)
Ex-smoker	390 520 (14.2)	439 503 (16.4)	257 087 (16.2)
Light (<10 cigarettes/day)	135 272 (4.9)	125 229 (4.7)	94 400 (6.0)
Moderate (10-19 cigarettes/day)	188 078 (6.8)	190 990 (7.1)	113 757 (7.2)
Heavy (>10 cigarettes/day)	107 288 (3.9)	158 134 (5.9)	86 787 (5.5)
Current smoking amount not recorded	43 957 (1.6)	78 372 (2.9)	65 106 (4.1)
Not recorded	780 226 (26.8)	963 580 (33.0)	193 038 (12.2)
Alcohol consumption:			
None	570 900 (20.8)	317 208 (11.8)	330 695 (20.9)
<1 unit/day	854 476 (31.1)	548 761 (20.4)	402 847 (25.4)
1-2 units/day	561 603 (20.4)	669 776 (24.9)	287 441 (18.2)
3-6 units/day	52 785 (1.9)	224 507 (8.4)	84 478 (5.3)
7-9 units/day	5750 (0.2)	38 273 (1.4)	8743 (0.6)
>9 units/day	2993 (0.1)	9583 (0.7)	7429 (0.5)
Not recorded	698 902 (25.4)	866 622 (32.3)	461 740 (29.2)
Previous major osteoporotic fracture	152 417 (5.5)	113 520 (4.2)	27 907 (1.8)
Parental history of osteoporosis or hip fracture	10 561 (0.4)	1077 (0.0004)	4227 (0.3)
Nursing or care home resident	16 819 (0.6)	7455 (0.3)	1535 (0.1)
Condition or prescription:			
Type 1 diabetes	8747 (0.3)	12 008 (0.4)	4322 (0.3)
Type 2 diabetes	81 715 (3.0)	100 009 (3.7)	43 437 (2.7)
History of falls	153 841 (5.6)	74 368 (2.8)	17 382 (1.1)
Dementia	34 892 (1.3)	15 036 (0.6)	7791 (0.5)
Cancer	94 090 (3.4)	67 380 (2.5)	28 203 (1.8)
Asthma or COPD	355 014 (12.9)	303 541 (11.3)	113 175 (7.1)
Cardiovascular disease	156 577 (5.7)	195 378 (7.3)	77 824 (4.9)
Chronic liver disease	6093 (0.2)	6753 (0.3)	3216 (0.2)
Chronic renal disease	33 274 (1.2)	24 395 (0.9)	3413 (0.2)
Parkinson’s disease	7585 (0.3)	8348 (0.3)	3650 (0.2)
Rheumatoid arthritis or SLE	11 970 (0.4)	32 950 (1.2)	10 091 (0.6)
Malabsorption	34 884 (1.3)	27 122 (1.0)	8026 (0.5)
Endocrine disorders	25 089 (0.9)	5866 (0.2)	7882 (0.5)
Epilepsy or prescribed anticonvulsants	66 145 (2.4)	59 214 (2.2)	26 271 (1.7)
Prescribed antidepressants	66 145 (2.4)	59 214 (2.2)	111 229 (7.0)
Prescribed corticosteroids	37 169 (1.4)	22 632 (0.8)	30 998 (2.0)
Prescribed oestrogen only HRT	33 679 (1.2)	127 (0.0)	14 988 (0.9)

Data are number (%) of participants unless stated otherwise.

SD=standard deviation; COPD=chronic obstructive pulmonary disease; HRT=hormone replacement therapy; SLE=systemic lupus erythematosus.

*Only whole population reported so could not be grouped by sex.

The crude incidence of both major osteoporotic fracture and hip fracture was higher in women than in men (major osteoporotic fracture 6.12 per 1000 person years in women *v* 2.26 in men; hip fracture 2.30 *v* 0.88, respectively) (online supplemental tables S7 and S8). We found a marked increase with age for both outcomes, and differences between the sexes were larger in older ages (eg, in women aged 30-34 years, major osteoporotic fracture was 0.95 per 1000 person years, increasing to 33.53 for ages 80-99 years; in men aged 30-34 years, 1.02 per 1000 person years increasing to 15.42 for ages 80-99 years) (online supplemental tables S9 and S10). For the whole population, the incidence of major osteoporotic fracture in this study was 4.22 per 1000 person years of follow-up compared with 2.45 per 1000 person years in the previously published updated QFracture internal validation cohort,^12^ and 2.89 per 1000 person years in a previously published Clinical Practice Research Datalink validation cohort.^12^ For hip fracture, overall incidence was 1.60 per 1000 person years compared with 1.32 in the same previously published Clinical Practice Research Datalink validation cohort.^28^ Two thirds (64 163, 67.1%) of major osteoporotic fractures in women and half (17 276, 50.3%) in men were in people aged ≥65 years. For hip fracture, 32 339 (88.8%) fractures in women and 10 167 (76.0%) in men were in people aged ≥65 years (online supplemental tables S7 and S8).

**Figure 1:**
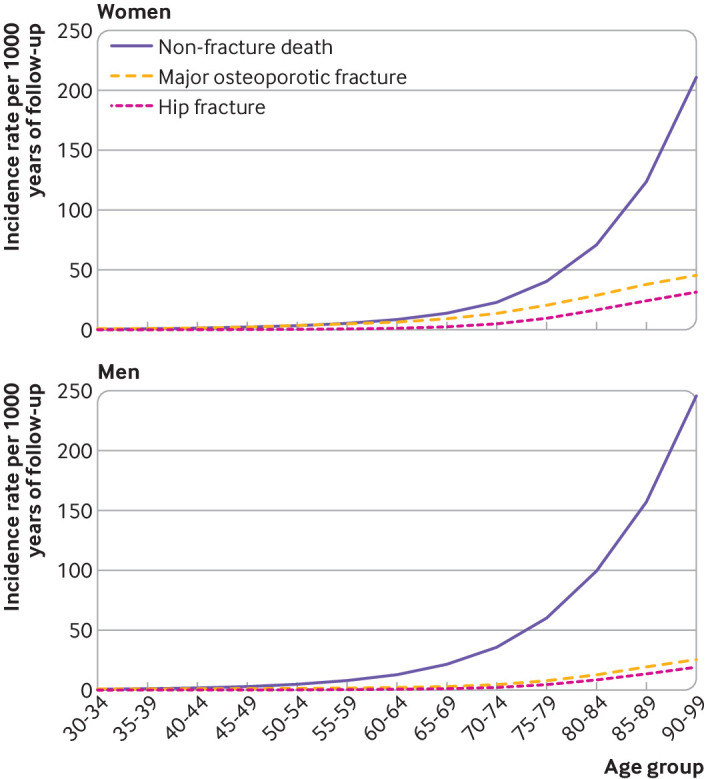
Incidence of major osteoporotic fracture, hip fracture, and death from causes other than fractures (non-fracture death) in women and men

Although the incidence of major osteoporotic fracture and hip fracture increased with age in men and women, the incidence of mortality from causes other than fractures increased more steeply with age (particularly in men). The incidence of death from causes other than fractures was similar to the incidence of major osteoporotic fracture in young people, but increased greatly with age; four times as common as major osteoporotic fracture in women aged 90-99 years and almost 10 times as common in men aged 90-99 years (figure 1, online supplemental tables S15 and S16). The incidence of death from causes other than fractures was higher than for hip fracture at all ages.

In the whole population, QFracture discrimination for major osteoporotic fracture was excellent in women (C=0.813) and good in men (C=0.738), and for hip fracture was excellent in both sexes (women C=0.918, men C=0.888) (table 2). Grouped by age, however, for both outcomes discrimination was poor to moderate in older adults where prediction of fracture risk is recommended^1^ (eg, for major osteoporotic fracture, ages 65-74 years, C=0.616 for women and 0.660 for men; ages 85-99 years, C=0.576 for women and C=0.624 for men) (table 2). Grouped by Charlson comorbidity index, discrimination was good for major osteoporotic fracture and good to excellent for hip fracture in all groups.

**Table 2:** Discrimination and model fit for major osteoporotic fracture and hip fracture*

	Women			Men		
	Harrell’s C	D statistic	R^2^	Harrell’s C	D index	R^2^
**Major osteoporotic fracture**						
All patients	0.813 (0.811 to 0.815)	2.25 (2.24 to 2.27)	54.8 (54.5 to 55.1)	0.738 (0.735 to 0.741)	1.76 (1.74 to 1.78)	42.4 (41.9 to 43.0)
Age group (years):						
30-64	0.709 (0.706 to 0.712)	1.30 (1.28 to 1.32)	28.8 (28.2 to 29.4)	0.625 (0.621 to 0.630)	0.84 (0.81 to 0.86)	14.4 (13.6 to 15.1)
65-74	0.616 (0.612 to 0.620)	0.71 (0.69 to 0.73)	10.7 (10.1 to 11.4)	0.660 (0.653 to 0.668)	1.00 (0.95 to 1.04)	19.2 (17.9 to 20.6)
75-84	0.615 (0.612 to 0.619)	0.67 (0.65 to 0.69)	9.6 (9.1 to 10.2)	0.652 (0.645 to 0.659)	0.91 (0.87 to 0.95)	16.4 (15.2 to 17.6)
85-99	0.576 (0.570 to 0.581)	0.38 (0.35 to 0.42)	3.4 (2.9 to 4.0)	0.624 (0.613 to 0.636)	0.67 (0.60 to 0.73)	9.6 (8.0 to 11.3)
Charlson comorbidity index:						
0	0.795 (0.793 to 0.798)	2.08 (2.06 to 2.10)	50.8 (50.4 to 51.2)	0.668 (0.664 to 0.673)	1.22 (1.20 to 1.25)	26.3 (25.4 to 27.1)
1	0.801 (0.797 to 0.805)	2.08 (2.05 to 2.10)	50.7 (50.1 to 51.4)	0.730 (0.723 to 0.737)	1.64 (1.59 to 1.68)	39.0 (37.7 to 40.2)
2	0.747 (0.742 to 0.753)	1.60 (1.56 to 1.63)	37.8 (36.9 to 38.8)	0.727 (0.719 to 0.736)	1.54 (1.49 to 1.60)	36.3 (34.6 to 37.9)
≥3	0.712 (0.706 to 0.718)	1.30 (1.26 to 1.33)	28.7 (27.5 to 29.8)	0.724 (0.715 to 0.733)	1.46 (1.40 to 1.51)	33.7 (32.0 to 35.4)
**Hip fracture**						
All patients	0.918 (0.915 to 0.921)	3.26 (3.24 to 3.28)	71.7 (71.4 to 71.9)	0.888 (0.882 to 0.893)	3.19 (3.16 to 3.23)	70.9 (70.4 to 71.3)
Age group (years):						
30-64	0.832 (0.823 to 0.841)	2.24 (2.19 to 2.30)	54.6 (53.4 to 55.8)	0.765 (0.755 to 0.776)	1.88 (1.82 to 1.94)	45.8 (44.1 to 47.4)
65-74	0.694 (0.687 to 0.701)	1.20 (1.16 to 1.24)	25.7 (24.4 to 27.0)	0.705 (0.694 to 0.716)	1.29 (1.23 to 1.36)	28.5 (26.5 to 30.5)
75-84	0.664 (0.659 to 0.669)	0.95 (0.92 to 0.98)	17.7 (16.8 to 18.5)	0.679 (0.670 to 0.687)	1.08 (1.03 to 1.13)	21.7 (20.1 to 23.3)
85-99	0.601 (0.595 to 0.608)	0.51 (0.47 to 0.55)	5.8 (5.0 to 6.7)	0.637 (0.623 to 0.651)	0.75 (0.67 to 0.82)	11.8 (9.8 to 13.9)
Charlson comorbidity index:						
0	0.924 (0.919 to 0.929)	3.36 (3.33 to 3.39)	72.9 (72.6 to 73.3)	0.852 (0.844 to 0.860)	2.84 (2.79 to 2.89)	65.8 (64.9 to 66.6)
1	0.899 (0.893 to 0.905)	2.92 (2.88 to 2.96)	67.1 (66.4 to 67.7)	0.872 (0.861 to 0.882)	2.89 (2.82 to 2.96)	66.7 (65.6 to 67.7)
2	0.839 (0.831 to 0.846)	2.24 (2.19 to 2.29)	54.5 (53.4 to 55.5)	0.808 (0.796 to 0.821)	2.17 (2.09 to 2.25)	53.0 (51.1 to 54.7)
≥3	0.783 (0.775 to 0.792)	1.75 (1.70 to 1.80)	42.2 (40.8 to 43.5)	0.782 (0.770 to 0.794)	1.90 (1.83 to 1.97)	46.4 (44.5 to 48.2)

Values are mean (95% confidence interval).

*Harrell’s C has values from 0.5 (no better than chance) to 1 (perfect discrimination). For the D statistic, higher values indicate better discrimination, and a difference of >0.1 has been proposed as indicating a meaningful difference in discrimination.^25^ R^2^ has values from 0 (no variation in the outcome is explained by the risk model) to 100% (the risk model explains all variation in the outcome).

**Figure 2:**
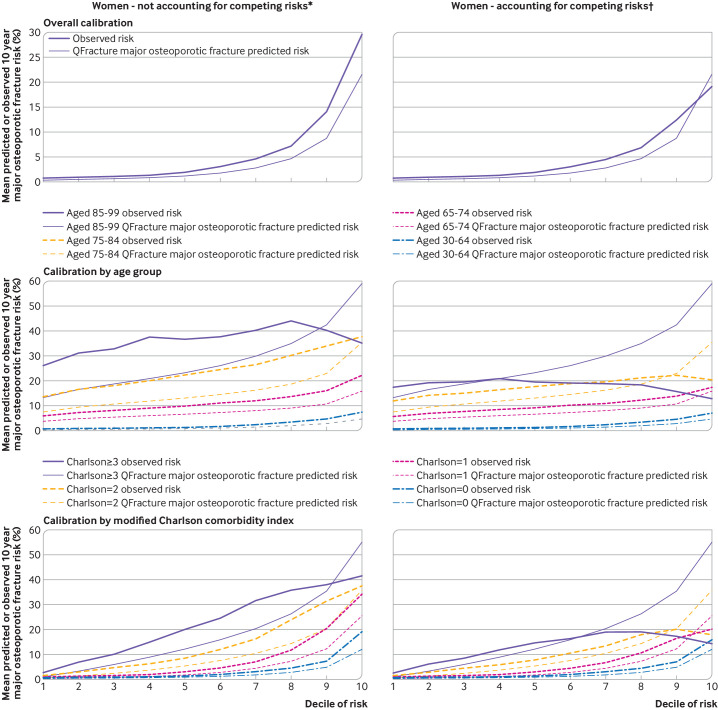
Calibration for major osteoporotic fracture in women without accounting for competing risks and accounting for competing risks. For each pair, observed risk curve above predicted risk curve indicates under-prediction; observed risk curve below predicted risk curve indicates over-prediction. Separate plots for age and Charlson comorbidity index are shown in supplementary figures S2 and S4, respectively. *Observed risk based on Kaplan-Meier estimator, which does not account for competing mortality risk. †Observed risk based on Aalen-Johansen estimator, which accounts for competing mortality risk

**Figure 3:**
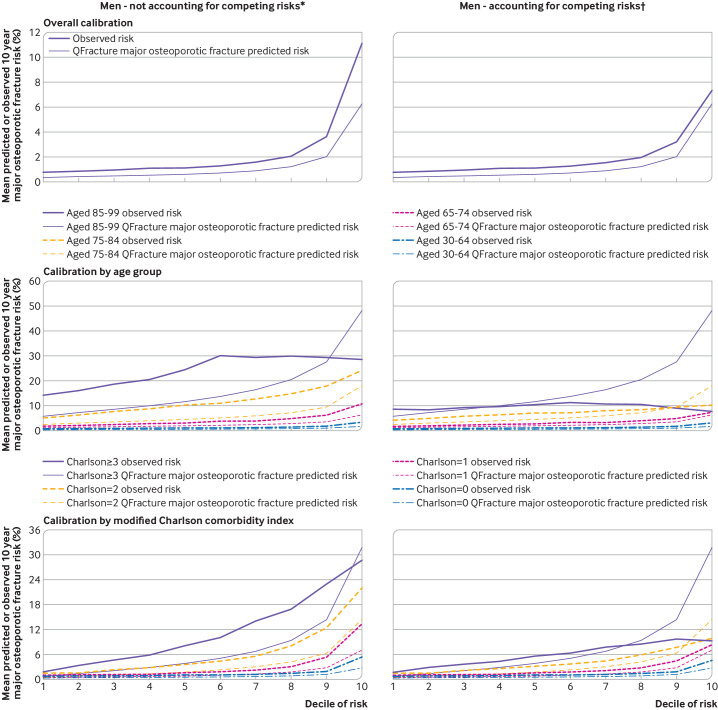
Calibration for major osteoporotic fracture in men without accounting for competing risks and accounting for competing risks. For each pair, observed risk curve above predicted risk curve indicates under-prediction; observed risk curve below predicted risk curve indicates over-prediction. Separate plots for age and Charlson comorbidity index are shown in supplementary figures S3 and S5, respectively. *Observed risk based on Kaplan-Meier estimator, which does not account for competing mortality risk. †Observed risk based on Aalen-Johansen estimator, which accounts for competing mortality risk

**Figure 4:**
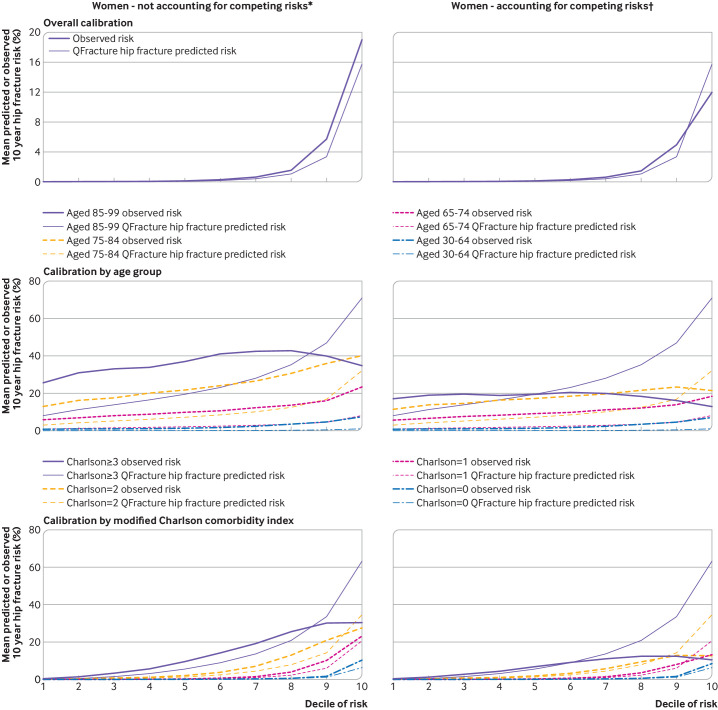
Calibration for hip fracture in women without accounting for competing risks and accounting for competing risks. For each pair, observed risk curve above predicted risk curve indicates under-prediction; observed risk curve below predicted risk curve indicates over-prediction. Separate plots for age and Charlson comorbidity index are shown in supplementary figures S6 and S8, respectively. *Observed risk based on Kaplan-Meier estimator, which does not account for competing mortality risk. †Observed risk based on Aalen-Johansen estimator, which accounts for competing mortality risk

Figures 2–4 and online supplemental figures S2–S9 show the calibration plots. When observed rates for major osteoporotic fracture were estimated without accounting for competing risk (figures 2 and 3 and online supplemental figures S2–S5), in the whole population for both men and women, we found under-prediction of the risk of fracture at all levels of predicted risk. Grouped by age, under-prediction in all age groups and at all levels of predicted risk was found except in the highest predicted risk decile in people aged 80-99 years where over-prediction was evident. Similar patterns were seen when grouped by Charlson comorbidity index, with under-prediction in all groups except those with the most multimorbidities at the highest levels of predicted risk.

**Figure 5:**
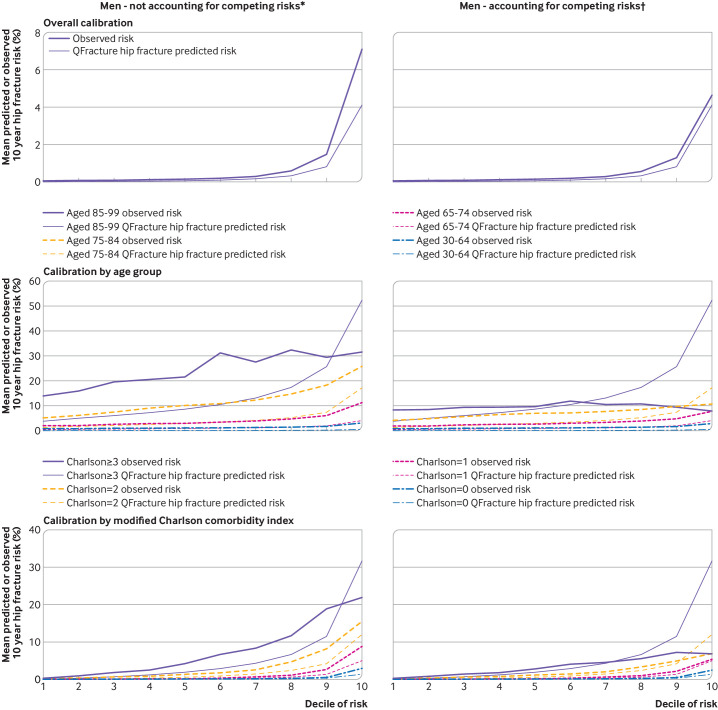
Calibration for hip fracture in men without accounting for competing risks and accounting for competing risks. For each pair, observed risk curve above predicted risk curve indicates under-prediction; observed risk curve below predicted risk curve indicates over-prediction. Separate plots for age and Charlson comorbidity index are shown in supplementary figures S7 and S9, respectively. *Observed risk based on Kaplan-Meier estimator, which does not account for competing mortality risk. †Observed risk based on Aalen-Johansen estimator, which accounts for competing mortality risk

When observed major osteoporotic fracture rates were estimated accounting for competing risk (figures 2 and 3 and online supplemental figures S2–S5), in the whole population, we found less under-prediction with some over-prediction in women at the highest predicted risk. Grouped by age, under-prediction was found in younger age groups but to a lesser extent than without accounting for competing risk. We found considerable over-prediction in women aged 85-99 years at higher risk and in most men aged 85-99 years, and over-prediction in men and women aged 75-84 years at the highest levels of predicted risk. In these older age groups, observed risk of major osteoporotic fracture was either flat or decreased as the decile of predicted risk increased. Similar patterns were seen when grouped by Charlson comorbidity index, with over-prediction of the risk of fracture in those with the most multimorbidities (Charlson comorbidity index ≥3) and in people with a Charlson comorbidity index of 2 at the highest level of predicted risk.

For hip fracture, when observed rates of hip fracture were estimated without accounting for competing risk (figures 4 and 5 and online supplemental figures S6–S9), in the whole population, we found greater under-prediction of the risk of fracture than for major osteoporotic fracture at all levels of predicted risk for both women and men. Grouped by age, we found under-prediction in all age groups and at all levels of predicted risk except for the highest two predicted risk deciles in women aged 80-99 years where large over-prediction of risk was found. Similar over-prediction was found in the highest risk decile for men aged 80-99 years. When grouped by Charlson comorbidity index, similar patterns were seen, with under-prediction in all groups except for those with the most multimorbidities at the highest levels of predicted risk.

When observed hip fracture rates were estimated accounting for competing risk (figures 4 and 5 and online supplemental figures S6–S9), in the whole population, we found less under-prediction with some over-prediction in women at the highest predicted risk. Grouped by age, under-prediction was less in younger age groups, but over-prediction was considerable in both sexes aged 85-99 years at higher predicted risk, as well as in both sexes aged 75-84 years at the highest levels of predicted risk. Similar to major osteoporotic fracture, in these two older age groups, observed hip fracture rates were flat or declined across all 10 deciles of increasing predicted risk. Similar patterns were seen when grouped by Charlson comorbidity index, with over-prediction of fracture risk in those with the most multimorbidities (Charlson comorbidity index ≥3) and in people with a Charlson comorbidity index of 2 at the highest level of predicted risk.

## Discussion

### Summary of findings

In this external validation of the QFracture risk prediction tool, we found very good to excellent discrimination in the whole population aged 30-99 years, but poor to good discrimination in important subgroups, including older patients and those with higher levels of multimorbidity. In contrast, calibration was poor. When evaluated without accounting for competing risk, QFracture consistently under-predicted both major osteoporotic fracture and hip fracture. The most likely explanation for this finding is that our method of determining the number of fractures in this study was more complete because fractures recorded during admission to hospital were included as well as those recorded in general practice electronic health records and death registrations. In this study, in women, only 14 802 (13.5%) major osteoporotic fractures and 6911 (19.0%) hip fractures were recorded in hospital admission data, compared with 6305 (18.4%) major osteoporotic fractures and 2515 (19.1%) hip fractures in men. Restricting determination of fractures to general practice and mortality data (to match the previously published internal^12^ and external validation studies^9 28^), largely explains the higher observed incidence of hip fracture in this study, but only partially explains the observed incidence of major osteoporotic fracture (online supplemental tables S11–S14, online supplemental figure S1). Also, the earliest study entry year in our study was 2004 compared with 1998 in the QFracture derivation, and recording of fractures in general practice data is likely to have improved over time.

When evaluated against observed fractures, estimated accounting for competing risk of mortality, under-prediction in general declined (because failing to account for competing risk causes over-prediction) but we found large over-prediction at higher levels of predicted risk in older people and in people with more complex multimorbidities. In people aged 85-99 years and in those with a Charlson comorbidity index of ≥3, observed risk was flat or even declining across deciles of increasing predicted risk. QFracture under-predicted in all patients because derivation was based on incomplete determination of fractures, and it over-predicted in people with a high competing risk of death (mainly elderly people and those with multiple comorbidities).

### Strengths and limitations

The strengths of the study include the use of linked population data, the conduct of the study in accordance with methodology recommendations,^24 29^ the codelists used all being published in the supplementary material to allow our findings to be replicated, the consideration of performance in important subgroups, and by accounting for competing risks of mortality. The high prevalence of missing data for some predictors was an important limitation, and a problem common to all studies that use routine data. Considering that QFracture used information recorded after participant study entry for some variables whereas we did not, more missing data for body mass index and smoking existed in this study compared with the QFracture internal derivation, although missingness (ie, the extent of missing data) for alcohol status and ethnic group was similar (online supplemental table S6). We used multiple imputation based on the assumption that data are missing at random, which is likely reasonable for the imputed variables in this context. Also, censoring is common with a median follow-up of 5-6 years in this study, similar to others that have used these types of data,^9 15^ including the QFracture derivation and validation studies.^8 9 12^ Although we explicitly accounted for censoring because of death in this study, our analysis, similar to others that have used these types of data, still assumes that people who deregister from a Clinical Practice Research Datalink practice have the same risk of fracture as those who do not. This assumption is likely strong in older people where deregistration because they moved into care housing, or to a nursing home or care home, might be associated with a higher risk of fracture. Studies that can continue to follow up participants even if they move practice would allow this assumption to be examined, which is increasingly possible with the expansion of data linkage driven by the covid-19 pandemic.

A further limitation of our study was that humeral fractures in general practice data are often recorded without specifying whether the fracture was proximal or more distal. Therefore, we defined humeral fractures as proximal if the site was not specifiied, which might have caused some misclassifications (some false positives). Most humeral fractures are proximal,^30^ however, and only including humeral fractures specified as proximal would have caused greater misclassification (many false negatives). We also could not validate our fractures against the gold standard of manually searching medical records, but our observed rates for hip fracture were similar to registry data.^30^ Finally, the QFracture prediction tool does not include data on bone mineral density because these data are not routinely available, and also one of the guideline recommended uses of the tool is to identify those who would benefit from measurement of bone mineral density. Including bone mineral density in the prediction would be expected to improve predictive performance, but investigating this effect was outside the scope of our analysis.

### Comparison with other literature

The first version of QFracture^8^ was independently externally validated in a similar dataset to ours (The Health Improvement Network) and found to have excellent discrimination and calibration in the whole population.^9^ The updated version of QFracture (evaluated in this study)^12^ was externally validated in the Clinical Practice Research Datalink by the QFracture derivation team who found excellent discrimination and calibration in the whole population.^28^ In this study, discrimination in the whole population for major osteoporotic fracture and hip fracture was similarly excellent. Given the large differences in the incidence of fractures across the age ranges studied, however, any prediction tool where the whole population includes people aged 30-99 years will have excellent discrimination.^31 32^ When grouped by age, discrimination varied from poor to moderate (as expected when the most powerful predictor of fracture is partially removed by examining age subgroups).^31 32^ Unlike these previously published validations in UK data,^8 9 12^ calibration was poor.

This study differs from previously published validations in two ways. Firstly, we also included fractures recorded during hospital admission (as well as those recorded in primary care electronic health records and in mortality data), and the primary care data were more recent and therefore recording of fractures in the general practitioner record might also have improved. Better determination of fractures would be expected to result in under-prediction by QFracture, as observed in this study. Consistent with this finding, an Israeli external validation based on community and hospital data for fractures also observed considerable under-prediction by QFracture.^7^ Because the codelists used by QFracture and in previous validations are unpublished, however, we cannot examine the extent to which differences relate to the choice of which fracture codes to include. Secondly, we examined calibration against observed outcomes estimated in the same way as previous external validations (with the Kaplan-Meier estimator, which does not account for competing mortality risk) and also accounting for competing risk (with the Aalen-Johansen estimator). As expected,^14 16 31^ when accounting for competing risks, large changes in observed risk in older people and those with more multimorbidities were found where death from causes other than fractures is more common, consistent with over-prediction by QFracture in people with a high competing mortality risk (despite under-prediction in all patients because of incomplete determination of fracture in the QFracture derivation).

### Implications for policy, practice, and research

QFracture and similar clinical prediction tools^28^ including a wide age range typically have excellent discrimination, but that likely reflects that age is a powerful predictor of most outcomes.^31 32^ As we found in this study, excellent discrimination in the whole population is compatible with poor discrimination and poor calibration in the subgroups most at risk of the outcome (older people and those with multiply morbidities). Examination of discrimination and calibration grouped by age (and other important predictors where applicable) provides a better indication of predictive performance from a clinical perspective. Future research could examine whether fracture prediction models that are more tailored to different age groups (including premenopausal and postmenopausal groups in women) provide better prediction (eg, osteoporosis might dominate the risk of fracture in younger people, whereas the risk of falls might be important in older people).

QFracture in its current form has two major problems. Firstly, this study and a previous external validation^7^ in Israel found that it under-predicts risk in general, most likely because its derivation is based on incomplete determination of fractures. This issue could be resolved by recalibration of the existing QFracture tool or derivation of a new version with better determination of fractures. Secondly, QFracture does not account for competing mortality risks that results in considerable over-prediction in people at high risk of death from other causes, notably older people and those with high level multimorbidities. Similar over-prediction has been observed in cardiovascular risk prediction models^15 33 34^ but the effect is greater for prediction of the risk of fracture because death related to fractures is a smaller proportion of total mortality than cardiovascular disease. This problem could be resolved by derivation of new models that explicitly account for competing risk. Both of these problems are resolvable, and we emphasise that the problem is not with the fracture risk prediction itself, but with the particular implementation of the current version of QFracture.

The FRAX fracture risk prediction tool is also recommended by NICE and accounts for competing risk of mortality, but systematic external validation is not possible because the prediction algorithm is not publicly available.^6 10^ Dagan et al reported an external validation of FRAX from primary and secondary care Israeli data, and found similar levels of under-prediction to QFracture (although their analysis did not account for competing risk of mortality).^7^ FRAX risk prediction was only approximately based on the number of clinical risk factors, however, rather than based on the actual FRAX risk equation because the FRAX prediction algorithm has never been made publicly available and therefore cannot be replicated. How FRAX accounts for competing risk of mortality and its performance in external validation is uncertain. Publication of the full algorithm would allow direct and fair comparison with other tools to identify the optimal tool for different contexts.^7^

There are implications for determining clinical risk and for decision making by patients and clinicians. Bisphosphonates are cost effective at relatively low thresholds of predicted risk^1^ but misclassification might occur with poor calibration. Consideration of the expected benefit for the individual is recommended in decision making, but aids to patient decision making usually rely on reasonably accurate prediction of individual risk.^35^ From this perspective, determining risk with the current version of QFracture will under-predict the risk of fracture in younger people and in those with less multimorbidities (and therefore underestimate the expected benefit of treatment) and will over-predict the risk of fracture in older people and those with high levels of multimorbidities (and will therefore overestimate expected benefit of treatment).

Therefore, new fracture risk prediction models, based on data with better determination of outcomes and that account for competing risk of mortality, need to be derived, internally validated, and externally validated. Equally, prediction in elderly people requires specific attention, building on small existing studies of prediction in this population.^36^ Updating the FRAX model, which accounts for competing mortality,^37^ is planned, but publication of the prediction algorithm will be critical in establishing its external validity.^24^

### Conclusion

This study found that QFracture under-predicts fracture risk in general because its derivation is based on incomplete determination of fractures, and considerably over-predicts in groups with a high risk of death from other causes because it does not account for competing mortality risk. Its use in clinical practice therefore needs review, particularly in people at high risk of death from other causes.

## Data Availability

Data may be obtained from a third party and are not publicly available. The data controller is the Clinical Practice Research Datalink (CPRD), and under the data licence granted, the authors are not allowed to share data. Researchers can apply to CPRD directly for access to the raw data.
