# Supplementary material for: Effect of competing mortality risks on predictive performance of the QFracture risk prediction tool for major osteoporotic fracture and hip fracture: external validation cohort study in a UK primary care population
Source: BMJ Med. 2022 Oct 25;1(1):e000316. doi: 10.1136/bmjmed-2022-000316 (PMC9978756; doi:10.1136/bmjmed-2022-000316)
Supplement: Supplementary data [file bmjmed-2022-000316supp001.pdf]

Impact of competing mortality risks on predictive performance of the QFracture risk prediction tool for major osteoporotic fracture and hip fracture: external validation cohort study in a UK primary care population

Supplementary file

Table S1: Read codes defining major osteoporotic fracture including hip fracture 3

Table S2: ICD-10 codes defining major osteoporotic fractures causing hospital admission 6

Table S3: Definitions of morbidity predictors for QFracture algorithm 7

Table S4: Read codes defining morbidity predictors (codesets created for this study) 8

Table S5: CPRD Procodes defining prescribing variables (corticosteroids are only oral or injectable preparations) 14

Table S6: Missing data 19

Table S7: Crude incidence of major osteoporotic fracture (MOF) over 10 years of follow-up 20

Table S8: Crude incidence of hip fracture over 10 years of follow-up 21

Table S9: Comparison of major osteoporotic fracture (MOF) incidence in this study and previous external validation study[a 3] 22

Table S10: Comparison of hip fracture incidence in this study and previous external validation study[a 3] 23

Table S11: Crude incidence of major osteoporotic fracture (MOF) over 10 years of follow-up (with ascertainment restricted to GP and mortality data)... 24

Table S12: Comparison of major osteoporotic fracture incidence in this study with complete fracture ascertainment (GP, mortality and hospital admission data), previous external validation study, and in this study using ascertainment to match previous study (GP and mortality data only) [3] 25

Table S13: Crude incidence of hip fracture over 10 years of follow-up (with ascertainment restricted to GP and mortality data) 26

Table S14: Comparison of hip fracture incidence in this study with complete fracture ascertainment (GP, mortality and hospital admission data), previous external validation study, and in this study using ascertainment to match previous study (GP and mortality data only)[a 3] 27

Figure S1: Comparison of fracture incidence in this study (using GP, mortality and hospital admission data), previous external validation (using GP and ONS data but maximum age 85) and this study matched to previous external validation ascertainment (using GP and ONS data)......... 28

Table S15: Crude incidence of non-fracture death over 10 years of follow-up............................... 29

Livingstone SJ, *et al. BMJMED* 2022; 1:e000316. doi: 10.1136/bmjmed-2022-000316

Table S16: Comparison of major osteoporotic fracture (MOF), hip fracture and non-fracture death incidence (rate per 1000 person/years [95%CI]) 30
Figure S2: Calibration for major osteoporotic fracture in women by agegroup without accounting for competing risks (left hand) and accounting for competing risks (right hand). 31
Figure S3: Calibration for major osteoporotic fracture in men by agegroup without accounting for competing risks (left hand) and accounting for competing risks (right hand)........ 32
Figure S4: Calibration for major osteoporotic fracture in women by Charlson Score without accounting for competing risks (left hand) and accounting for competing risks (right hand) 33
Figure S5: Calibration for major osteoporotic fracture in men by Charlson Score without accounting for competing risks (left hand) and accounting for competing risks (right hand) 34
Figure S6: Calibration for hip fracture in women by agegroup without accounting for competing risks (left hand) and accounting for competing risks (right hand) 35
Figure S7: Calibration for hip fracture in men by agegroup without accounting for competing risks (left hand) and accounting for competing risks (right hand) 36
Figure S8: Calibration for hip fracture in women by Charlson Score without accounting for competing risks (left hand) and accounting for competing risks (right hand)........ 37
Figure S9: Calibration for hip fracture in men by Charlson Score without accounting for competing risks (left hand) and accounting for competing risks (right hand) 38

Table S1: Read codes defining major osteoporotic fracture including hip fracture

Fracture type	CPRD Medcode	Read Code	Read Code description
Hip	2225	S30..00	Fracture of neck of femur
Hip	1994	S30..11	Hip fracture
Hip	38489	S300.00	Closed fracture proximal femur, transcervical
Hip	39984	S300000	Cls # prox femur, intracapsular section, unspecified
Hip	69919	S300100	Closed fracture proximal femur, transepiphyseal
Hip	65690	S300200	Closed fracture proximal femur, midcervical section
Hip	52194	S300300	Closed fracture proximal femur, basicervical
Hip	51861	S300311	Closed fracture, base of neck of femur
Hip	36391	S300400	Closed fracture head of femur
Hip	17019	S300500	Cls # prox femur, subcapital, Garden grade unspec.
Hip	34351	S300600	Closed fracture proximal femur, subcapital, Garden grade I
Hip	33957	S300700	Closed fracture proximal femur, subcapital, Garden grade II
Hip	36599	S300800	Closed fracture proximal femur, subcapital, Garden grade III
Hip	34078	S300900	Closed fracture proximal femur, subcapital, Garden grade IV
Hip	49209	S300y00	Closed fracture proximal femur, other transcervical
Hip	68229	S300y11	Closed fracture of femur, subcapital
Hip	62966	S300z00	Closed fracture proximal femur, transcervical, NOS
Hip	5301	S302.00	Closed fracture of proximal femur, pertrochanteric
Hip	19117	S302000	Cls # proximal femur, trochanteric section, unspecified
Hip	19387	S302011	Closed fracture of femur, greater trochanter
Hip	48337	S302012	Closed fracture of femur, lesser trochanter
Hip	45141	S302100	Closed fracture proximal femur, intertrochanteric, two part
Hip	29145	S302200	Closed fracture proximal femur, subtrochanteric
Hip	51216	S302300	Cls # proximal femur, intertrochanteric, comminuted
Hip	8648	S302400	Closed fracture of femur, intertrochanteric
Hip	44735	S302z00	Cls # of proximal femur, pertrochanteric section, NOS
Hip	28965	S304.00	Pertrochanteric fracture
Hip	8243	S305.00	Subtrochanteric fracture
Hip	24276	S30w.00	Closed fracture of unspecified proximal femur
Hip	18273	S30y.00	Closed fracture of neck of femur NOS
Hip	10570	S30y.11	Hip fracture NOS
Hip	37662	S310000	Closed fracture of femur, unspecified part
Hip	520	S31z.00	Fracture of femur, NOS
Distal radius/ulna	5951	7K1LM00	Closed reduction of fracture of wrist
Distal radius/ulna	18299	S234.00	Closed fracture of radius and ulna, lower end
Distal radius/ulna	203	S234.11	Wrist fracture - closed
Distal radius/ulna	18389	S234000	Closed fracture of forearm, lower end, unspecified
Distal radius/ulna	343	S234100	Closed Colles' fracture
Distal radius/ulna	52389	S234111	Smith's fracture - closed
Distal radius/ulna	1742	S234200	Closed fracture of the distal radius, unspecified
Distal radius/ulna	28708	S234600	Closed fracture radius and ulna, distal
Distal radius/ulna	2862	S234700	Closed Smith's fracture

Livingstone SJ, *et al. BMJMED* 2022; 1:e000316. doi: 10.1136/bmjmed-2022-000316

Distal radius/ulna	40268	S234800	Closed Galeazzi fracture
Distal radius/ulna	11066	S234900	Closed volar Barton's fracture
Distal radius/ulna	53689	S234911	Closed volar Barton's fracture-dislocation
Distal radius/ulna	65636	S234912	Closed volar Barton fracture-subluxation
Distal radius/ulna	50053	S234A00	Closd dorsal Barton's fracture
Distal radius/ulna	57736	S234A11	Closed dorsal Barton's fracture-dislocation
Distal radius/ulna	107741	S234A12	Closed dorsal Barton fracture-subluxation
Distal radius/ulna	44844	S234C00	Closed fracture distal radius, intra-articular, die-punch
Distal radius/ulna	19058	S234D00	Closed fracture distal radius, extra-articular, other type
Distal radius/ulna	28293	S234E00	Closed fracture distal radius, intra-articular, other type
Distal radius/ulna	10033	S234F00	Closed Barton's fracture
Distal radius/ulna	102302	S234G00	Greenstick fracture of distal radius
Distal radius/ulna	27591	S234z00	Closed fracture of forearm, lower end, NOS
Distal radius/ulna	199	S23B.00	Fracture of lower end of radius
Distal radius/ulna	6213	S23C.00	Fracture of lower end of both ulna and radius
Distal radius/ulna	50654	S23x000	Closed fracture of forearm, unspecified
Distal radius/ulna	17952	S23x100	Closed fracture of radius (alone), unspecified
Distal radius/ulna	137	S23x111	Fracture of radius NOS
Distal radius/ulna	17922	S4C0000	Closed fracture-dislocation distal radio-ulnar joint
Distal radius/ulna	38408	S4C0100	Closed fracture-dislocation radiocarpal joint
Distal radius/ulna	44652	S4C2000	Closed fracture-subluxation, distal radio-ulnar jt
Distal radius/ulna	50148	S4C2100	Closed fracture-subluxation radiocarpal joint
Proximal humerus	6379	7K1LF00	Closed reduction of fracture of humerus
Proximal humerus	517	S22..00	Fracture of humerus
Proximal humerus	11222	S220.00	Closed fracture of the proximal humerus
Proximal humerus	44721	S220000	Closed fracture of proximal humerus, unspecified part
Proximal humerus	11313	S220100	Closed fracture proximal humerus, neck
Proximal humerus	33489	S220200	Closed fracture of proximal humerus, anatomical neck
Proximal humerus	11044	S220300	Closed fracture proximal humerus, greater tuberosity
Proximal humerus	28739	S220400	Closed fracture proximal humerus, head
Proximal humerus	52406	S220500	Closed fracture of humerus, upper epiphysis
Proximal humerus	40330	S220600	Closed fracture proximal humerus, three part
Proximal humerus	29137	S220700	Closed fracture proximal humerus, four part
Proximal humerus	38353	S220z00	Closed fracture of proximal humerus not otherwise specified
Proximal humerus	19186	S222000	Closed fracture of humerus NOS
Proximal humerus	2101	S226.00	Fracture of upper end of humerus
Proximal humerus	10382	S22z.00	Fracture of humerus NOS
Vertebral	16895	N1y1.00	Fatigue fracture of vertebra
Vertebral	44386	N331.14	Osteoporotic vertebral collapse
Vertebral	15837	N331011	Collapse of thoracic vertebra
Vertebral	17377	N331800	Osteoporosis + pathological fracture lumbar vertebrae
Vertebral	12673	N331900	Osteoporosis + pathological fracture thoracic vertebrae
Vertebral	48772	N331A00	Osteoporosis + pathological fracture cervical vertebrae
Vertebral	9319	N331F00	Collapse of thoracic vertebra
Vertebral	45736	N331H00	Collapse of cervical vertebra due to osteoporosis
Vertebral	5841	N331J00	Collapse of lumbar vertebra due to osteoporosis

Vertebral	19048	N331K00	Collapse of thoracic vertebra due to osteoporosis
Vertebral	4013	N331L00	Collapse of vertebra due to osteoporosis NOS
Vertebral	53337	S100H00	Closed fracture cervical vertebra, wedge
Vertebral	27404	S102.00	Closed fracture thoracic vertebra
Vertebral	28524	S102100	Closed fracture thoracic vertebra, wedge
Vertebral	8266	S104100	Closed fracture lumbar vertebra, wedge
Vertebral	5381	S15..00	Fracture of thoracic vertebra

Table S2: ICD-10 codes defining major osteoporotic fractures causing hospital admission

**Fracture type**	**ICD10-code**	**ICD-10 Code description**
Hip	S72.0	Fracture of neck of femur
Hip	S72.1	Pertrochanteric fracture
Hip	S72.2	Subtrochanteric fracture
Distal radius/ulna	S52.5	Fracture of lower end of radius
Distal radius/ulna	S52.6	Fracture of lower end of both ulna and radius
Proximal humerus	S42.2	Fracture of upper end of humerus
Vertebral	M48.5	Collapsed vertebra, not elsewhere classified
Osteoporotic	M80.0	Postmenopausal osteoporosis with pathological fracture
Osteoporotic	M80.1	Postoophorectomy osteoporosis with pathological fracture
Osteoporotic	M80.3	Postsurgical malabsorption osteoporosis with pathological fracture
Osteoporotic	M80.5	Idiopathic osteoporosis with pathological fracture
Osteoporotic	M80.8	Other osteoporosis with pathological fracture
Osteoporotic	M80.9	Unspecified osteoporosis with pathological fracture

Table S3: Definitions of morbidity predictors for QFracture algorithm

**Morbidity**	**How defined**
Type 1 and type 2 diabetes	As defined for GP data in Kuan *et al* (2019)[1]
Parental history of osteoporosis/hip fracture	Bespoke codeset (table SX)
Care home resident	Bespoke codeset (table SX)
Previous fracture	As per fracture outcomes (table SX) plus bespoke codeset for 'history of' codes (table SX)
History of falls	Bespoke codeset (table SX)
Dementia	As defined for GP data in Kuan *et al* (2019)[1]
Cancer	As defined for GP data in Kuan *et al* (2019)[1]
Asthma or COPD	As defined for GP data in Kuan *et al* (2019)[1]
Heart attack, angina, stroke or TIA	CVD outcomes in GP data defined in supplementary file at Livingstone *et al* (2021)[2]
Chronic liver disease	As defined for GP data in Kuan *et al* (2019)[1]
Chronic kidney disease	As defined for GP data in Kuan *et al* (2019)[1]
Parkinson's Disease	As defined for GP data in Kuan *et al* (2019)[1]
Rheumatoid arthritis or SLE	As defined in GP data in Kuan *et al* (2019)[1]
Malabsorption[a]	Crohn's Disease, ulcerative colitis and coeliac disease as defined for GP data in Kuan *et al* (2019)[1]; malabsorption, steatorrhoea or blind loop syndrome bespoke codeset (table SX)
Endocrine problems[b]	Hyperparathyroidism as defined for GP data in Kuan *et al* (2019)[1], thyrotoxicosis and Cushing syndrome bespoke codeset (table SX)
Epilepsy	As defined for GP data in Kuan *et al* (2019)[1]

a. Crohn's disease, ulcerative colitis, coeliac disease, steatorrhoea or blind loop syndrome

b. Thyrotoxicosis, hyperparathyroidism, Cushing syndrome

Table S4: Read codes defining morbidity predictors (codesets created for this study)

Morbidity	CPRD Medcode	Read Code	Read Code description
Parental history of osteoporosis or hip fracture	11218	1268.00	FH: Osteoporosis
Family history of osteoporosis or hip fracture	51427	12I6.00	FH: Fragility fracture
Family history of osteoporosis or hip fracture	37204	12I4.00	FH: Maternal hip fracture
Family history of osteoporosis or hip fracture	42319	12I5.00	FH: Hip fracture in first degree relative
Family history of osteoporosis or hip fracture	43219	12I8.00	FH: maternal hip fracture before age 75
Care home resident	13359	13F6100	Lives in a nursing home
Care home resident	7653	9N1G.00	Seen in nursing home
Care home resident	24956	13FK.00	Lives in a residential home
Care home resident	13360	13F6.00	Nursing/other home
Care home resident	49681	13FX.00	Lives in care home
Care home resident	27968	13F7.00	Residential institution
Care home resident	13361	13F4.11	Lives in warden controlled accommodation
Care home resident	30807	13F4000	Resident in sheltered accommodation
Care home resident	98592	8Ce4.00	Preferred place of care - nursing home
Care home resident	6859	9N1F.00	Seen in warden sup home
Care home resident	93998	9b0i.00	Residential home visit note
Care home resident	10993	ZLG4.00	Discharge to nursing home
Care home resident	101003	9NFR.00	Home visit request by residential institution
Care home resident	28773	ZV60700	[V]Sheltered housing
Care home resident	100080	8Ce5.00	Preferred place of care - residential home
Care home resident	7101	9N1F.12	Seen in old people's home
Care home resident	59653	6991.00	Geriatric home admission exam.
Care home resident	73321	9b1P.00	Nursing home
Care home resident	102493	8Ht..00	Admission to nursing home
Care home resident	107443	9NFW000	Care home visit for initial patient assessment
Care home resident	35187	9N1D.00	Seen in warden sup house
Care home resident	35172	9N1E.00	Seen in warden sup flat
Care home resident	34794	13F9.11	Living in sheltered accomodatn
Care home resident	21280	13F5200	Resident in part III accomodation
Care home resident	107602	9NFW100	Care home visit for follow-up patient review
Care home resident	42191	ZLG3.00	Discharge to residential home
Care home resident	24828	Z177F00	Nursing home care
Care home resident	73083	9b0Y.00	Nursing home visit note
Care home resident	94070	8O24.00	Provision of continuing care in nursing home
Care home resident	107757	9NFW.00	Care home visit
Care home resident	59548	13FT.00	Lives in an old peoples home
Care home resident	102598	8Hs..00	Discharge to nursing home
Care home resident	27936	8HE6.00	Delayed discharge to nursing home
Care home resident	24816	Z177C00	Residential care
Care home resident	50792	9N1F.11	Seen in Part 3 accomodation

Care home resident	36096	13F5.11	Part 3 accomodation
Care home resident	6991	9493.00	Patient died in nursing home
Care home resident	43915	ZLG4100	Discharge to private nursing home
Care home resident	49138	ZV63212	[V]Delayed discharge - nursing home vacancy awaited
Care home resident	27360	13F5100	Part III accomodation arranged
Care home resident	98758	13Zo.00	Previously lived in care home
Care home resident	36905	ZLG5100	Discharge to warden controlled accommodation
Care home resident	35040	ZLG5.00	Discharge to sheltered housing
Care home resident	102230	M270100	Nursing home acquired pressure ulcer
Care home resident	48549	ZLG3100	Discharge to private residential home
Care home resident	95795	923O.00	FP22 - removal from residential institute
Care home resident	67903	U105100	[X]Fall involvng wheelchair occurrence residential instit'n
Care home resident	46642	9b79.00	Other residential care homes managed by local authority
Care home resident	66122	13F5111	Part 3 accommodation arranged
Care home resident	99148	9b7A.00	Other residential care home man voluntary/private agents
Care home resident	96836	ZK76.00	Temporary home care service provision
History of fracture[a]	17936	14G7.00	H/O: hip fracture
History of fracture[a]	18731	14G6.00	H/O: fragility fracture
History of fracture[a]	19235	14G8.00	H/O: vertebral fracture
History of falls	384	TC...11	Fall - accidental
History of falls	6815	TC...00	Accidental falls
History of falls	6008	16D..00	Falls
History of falls	4859	R200.12	[D] Geriatric fall
History of falls	6835	TCz..00	Accidental falls NOS
History of falls	8694	16D1.00	Recurrent falls
History of falls	8730	TCy..00	Other falls
History of falls	15112	TC5..00	Fall on same level from slipping, tripping or stumbling
History of falls	11307	TC0..00	Fall on or from stairs or steps
History of falls	11308	TCyz.00	Other accidental fall NOS
History of falls	17167	TC01.00	Fall on or from stairs
History of falls	11709	TC51.00	Fall on same level from tripping
History of falls	33887	TC4..00	Other fall from one level to another
History of falls	17728	TC01000	Fall on stairs
History of falls	108062	16D6.00	Fall
History of falls	18007	TC50.00	Fall on same level from slipping
History of falls	21081	TC01100	Fall from stairs
History of falls	26432	TC42100	Fall from bed
History of falls	7948	TC52.00	Fall on same level from stumbling
History of falls	33529	TC5z.00	Fall on same level from slipping
History of falls	98223	16D5.00	Fall onto outstretched hand
History of falls	41909	TC01z00	Fall on or from stairs NOS
History of falls	43092	TC02000	Fall on steps
History of falls	43571	TC3..00	Fall into hole or other opening in surface
History of falls	21306	TC4z.00	Fall from one level to another NOS

History of falls	53082	TC02100	Fall from steps
History of falls	44626	TC02.00	Fall on or from steps
History of falls	38818	TC42000	Fall from chair
History of falls	64696	TC0z.00	Fall on or from stairs or steps NOS
History of falls	7876	TC4yz00	Other fall from one level to another NOS
History of falls	41853	TC4y.00	Other fall from one level to another
History of falls	69020	TC4y200	Fall from stationary vehicle
History of falls	93574	8O9..00	Provision of telecare community alarm service
History of falls	53463	TC00.00	Fall on or from escalator
History of falls	56316	TC00000	Fall on escalator
History of falls	64722	TC02z00	Fall on or from steps NOS
History of falls	59404	TC42.00	Fall from chair or bed
History of falls	29568	TC3yz00	Fall into other hole
History of falls	55743	67ID.00	Falls advice - hip protectors advised
History of falls	48309	67IE.00	Falls advice - hip protectors supplied
History of falls	44119	8BIG.00	Falls caused by medication
History of falls	109088	9NIf.00	Seen by community falls team
History of falls	16684	T04..00	Fall in
History of falls	58753	T040.00	Fall in train
History of falls	94933	T040100	Fall in train
History of falls	59911	T041.00	Fall on train
History of falls	97335	T04z.00	Fall in
History of falls	18097	T170.00	MVTA - fall down stairs of motor bus while board/alighting
History of falls	41114	T171.00	MVTA - fall from car in street while boarding/alighting
History of falls	60782	T53..00	Fall in
History of falls	110413	T53z.00	Fall in
History of falls	60003	TC42z00	Fall from chair or bed NOS
History of falls	17638	TH03.00	Late effects of accidental fall
History of falls	7970	U10..00	[X]Falls
History of falls	21903	U100.00	[X]Fall on same level involving ice and snow
History of falls	68559	U100000	[X]Fall on same level involving ice and snow occurrn home
History of falls	63515	U100200	[X]Fall sam lvl inv ice/snw occ sch oth inst/pub admin area
History of falls	43615	U100300	[X]Fall same levl involv ice/snow
History of falls	60427	U100400	[X]Fall same levl inv ice and snow
History of falls	93148	U100500	[X]Fall same levl inv ice / snow
History of falls	71613	U100z00	[X]Fall same levl inv ice / snow
History of falls	29821	U101.00	[X]Fall on same level from slipping
History of falls	49035	U101000	[X]Fall same levl frm slip trip + stumb
History of falls	49210	U101100	[X]Fall same level from slip trip + stumb occ resid instit
History of falls	60424	U101200	[X]Fall sme levl slp trp+stmb occ sch
History of falls	49100	U101300	[X]Fall sme levl frm slip trip+stumb
History of falls	52452	U101400	[X]Fall same level from slip trip+stumb
History of falls	68616	U101500	[X]Fall sme lvl frm slip trip+stumb
History of falls	68895	U101600	[X]Fall same levl

History of falls	61705	U101700	[X]Fall same level from slip trip+stumbling
History of falls	49218	U101y00	[X]Fall same level
History of falls	68579	U101z00	[X]Fall same levl frm slip trip+stumbling
History of falls	111606	U102200	[X]Fall
History of falls	66934	U103000	[X]Oth fall same levl
History of falls	109428	U103500	[X]Oth fall sme levl coll/push anth pers occ trad/serv area
History of falls	62109	U103y00	[X]Oth fall sme levl coll/push anoth per occ oth spec place
History of falls	93454	U103z00	[X]Oth fall same levl coll/push anoth pers occ unspec place
History of falls	67230	U104.00	[X]Fall while being carried or supported by other persons
History of falls	52410	U104000	[X]Fall while carried/supported by other persons
History of falls	51851	U104100	[X]Fall whle carried/supported oth persons occ resid instit
History of falls	110968	U104z00	[X]Fall whle carr'd/supportd by oth per
History of falls	21349	U105.00	[X]Fall involving wheelchair
History of falls	98315	U105000	[X]Fall involving wheelchair
History of falls	67903	U105100	[X]Fall involvng wheelchair occurrence residential instit'n
History of falls	85959	U105500	[X]Fall involvng wheelchair occurrnce at trade/service area
History of falls	98713	U105700	[X]Fall involving wheelchair
History of falls	109423	U105y00	[X]Fall involv wheelchair
History of falls	52374	U106.00	[X]Fall involving bed
History of falls	44419	U106000	[X]Fall involving bed
History of falls	69762	U106100	[X]Fall involving bed occurrence in residential institution
History of falls	50572	U107.00	[X]Fall involving chair
History of falls	68600	U107000	[X]Fall involving chair
History of falls	68617	U107z00	[X]Fall involving chair
History of falls	55553	U108.00	[X]Fall involving other furniture
History of falls	68591	U108000	[X]Fall involving other furniture
History of falls	66922	U108100	[X]Fall involv other furniture occurrn resident institut'n
History of falls	36402	U10A.00	[X]Fall on and from stairs and steps
History of falls	52432	U10A000	[X]Fall on and from stairs and steps
History of falls	52466	U10A100	[X]Fall on + from stair + step occurrnce resident instit'n
History of falls	111571	U10A200	[X]Fall on + frm stair + step occ sch oth inst/pub adm area
History of falls	99385	U10A400	[X]Fall on + from stairs + steps occurrn on street/highway
History of falls	51284	U10A500	[X]Fall on + from stair + step occurrn at trade/servce area
History of falls	41105	U10A511	[X]Fall on or from escalator
History of falls	68613	U10Ay00	[X]Fall on + from stair + step occurrn at oth specif place
History of falls	64193	U10Az00	[X]Fall on + from stair + step occurrnce at unspecif place
History of falls	50316	U10D.00	[X]Fall from
History of falls	52380	U10D000	[X]Fall from out of/through building/structur occurn home
History of falls	100710	U10D100	[X]Fall from out of/thro buildng/struct occ resid instit'n

History of falls	110898	U10D400	[X]Fall from out/thro buildng/struct occ on street/highway
History of falls	92721	U10H.00	[X]Other fall from one level to another
History of falls	51669	U10H000	[X]Other fall from one level to another
History of falls	68609	U10H200	[X]Othr fall frm one level to anothr
History of falls	68562	U10H400	[X]Othr fall from one level to anothr occurrn street/h'way
History of falls	68604	U10H500	[X]Other fall frm one level to anothr occ at trde/serv area
History of falls	95961	U10H600	[X]Other fall frm one level to anoth occ indust/constr area
History of falls	72468	U10Hy00	[X]Other fall frm one levl to anothr occ at oth specif plce
History of falls	49233	U10Hz00	[X]Othr fall frm one level to anothr occurrn at unspec plce
History of falls	48496	U10J.00	[X]Other fall on same level
History of falls	43191	U10J000	[X]Other fall on same level
History of falls	72474	U10J100	[X]Other fall on same level
History of falls	100060	U10J200	[X]Other fall on same levl occ schl oth inst/pub admin area
History of falls	101254	U10J400	[X]Other fall on same level
History of falls	68608	U10J600	[X]Other fall on same levl
History of falls	101523	U10Jy00	[X]Other fall on same level occurrn at oth specified place
History of falls	98876	U10Jz00	[X]Other fall on same level occurrence at unspecified place
History of falls	24776	U10z.00	[X]Unspecified fall
History of falls	10419	U10z000	[X]Unspecified fall
History of falls	46303	U10z100	[X]Unspecified fall
History of falls	55202	U10z300	[X]Unspecified fall
History of falls	97327	U10z400	[X]Unspecified fall
History of falls	106900	U10z700	[X]Unspecified fall
History of falls	96546	U10zy00	[X]Unspecified fall
History of falls	61170	U10zz00	[X]Unspecified fall
History of falls	6785	ZV71B00	[V]Examination and observation following a fall
Malabsorption[b]	9355	J69..00	Intestinal malabsorption
Malabsorption[b]	5088	J69yz00	Other gastrointestinal tract malabsorption NOS
Malabsorption[b]	4787	J690.15	Steatorrhea - idiopathic
Malabsorption[b]	6663	J69y.00	Other intestinal malabsorption
Malabsorption[b]	42715	J69z.00	Intestinal malabsorption NOS
Malabsorption[b]	23498	J692.00	Blind loop syndrome
Malabsorption[b]	31392	J69y600	Intestinal malabsorption of fat
Malabsorption[b]	2482	D011100	Vit B12 defic anaemia due to malabsorption with proteinuria
Malabsorption[b]	19441	C285.00	Adult osteomalacia due to malabsorption
Malabsorption[b]	37440	J693.11	Postsurgical malabsorption - other
Malabsorption[b]	49191	J69y200	Intestinal malabsorption of protein
Malabsorption[b]	55481	D012300	Folate-deficiency anaemia due to malabsorption
Malabsorption[b]	72529	Jyu9000	[X]Other intestinal malabsorption
Malabsorption[b]	57647	J693100	Post gastrointestinal tract surgery malnutrition
Malabsorption[b]	49739	J69y300	Intestinal malabsorption of carbohydrate

Malabsorption[b]	93655	N330700	Postsurgical malabsorption osteoporosis
Endocrine problems[c]	1472	C02..11	Hyperthyroidism
Endocrine problems[c]	5257	C020.12	Graves' disease
Endocrine problems[c]	6245	1431.00	H/O: hyperthyroidism
Endocrine problems[c]	3857	C052.11	Autoimmune thyroiditis
Endocrine problems[c]	17604	C150.00	Cushing's syndrome
Endocrine problems[c]	11947	L181500	Postpartum thyroiditis
Endocrine problems[c]	30799	C051.00	Subacute thyroiditis
Endocrine problems[c]	4898	C050.00	Acute thyroiditis
Endocrine problems[c]	18382	C150111	Drug-induced Cushings syndrome
Endocrine problems[c]	26362	212P.00	Hyperthyroidism resolved
Endocrine problems[c]	106640	C025.00	Subclinical hyperthyroidism
Endocrine problems[c]	21747	C051.11	De Quervain's thyroiditis
Endocrine problems[c]	20275	C150100	Iatrogenic Cushing's syndrome
Endocrine problems[c]	60534	C150z00	Cushing's syndrome NOS
Endocrine problems[c]	49508	C024.00	Thyrotoxicosis from ectopic thyroid nodule
Endocrine problems[c]	68626	FyuBD00	[X]Dysthyroid exophthalmos
Endocrine problems[c]	42323	C050z00	Acute thyroiditis NOS
Endocrine problems[c]	53682	C150200	Pituitary dependent Cushing's syndrome
Endocrine problems[c]	65444	C05y.00	Other and unspecified chronic thyroiditis
Endocrine problems[c]	61026	C054.00	Iatrogenic thyroiditis
Endocrine problems[c]	53667	C053.11	Riedel's thyroiditis
Endocrine problems[c]	65907	C05y400	Chronic thyroiditis with transient thyrotoxicosis
Endocrine problems[c]	65754	C150500	Alcohol-induced pseudo-Cushing's syndrome
Endocrine problems[c]	67972	C050000	Acute nonsuppurative thyroiditis
Endocrine problems[c]	65120	C150300	Ectopic ACTH secretion causing Cushing's syndrome
Endocrine problems[c]	60690	F395100	Myopathy due to Cushing's syndrome
Endocrine problems[c]	70967	C150000	Idiopathic Cushing's syndrome
Endocrine problems[c]	56270	C024z00	Thyrotoxicosis from ectopic thyroid nodule NOS
Endocrine problems[c]	70773	C050100	Acute suppurative thyroiditis
Endocrine problems[c]	95807	Cyu4500	[X]Other Cushing's syndrome
Endocrine problems[c]	64656	C024000	Thyrotoxicosis from ectopic thyroid nodule with no crisis

a. Used along with fracture outcomes to define baseline history of fracture

b. Malabsorption includes Crohn's Disease, ulcerative colitis and coeliac disease as defined by Kuan *et al*[1] – these codes are for malabsorption, steatorrhoea or blind loop syndrome

c. Endocrine problems includes hyperparathyroidism as defined by Kuan *et al* (2019)[1] – these codes are for thyrotoxicosis and Cushing syndrome

Table S5: CPRD Procodes defining prescribing variables (corticosteroids are only oral or injectable preparations)

QFracture variable	CPRD drugsubstance (drug name as recorded in CPRD)	CPRD Prodcodes
Antidepressants	Amitriptyline hydrochloride	34916, 45242, 83, 33090, 52867, 24141, 57972, 55491, 70991, 61835, 76839, 45233, 34731, 66578, 80135, 57107, 65879, 24152, 59161, 34401, 46801, 64000, 79826, 70300, 76298, 46818, 76927, 487, 34197, 41729, 42394, 34474, 32439, 49, 34782, 54877, 24145, 55139, 42078, 71042, 65987, 64647, 79766, 34503, 24134, 66579, 60355, 77167, 65439, 66572, 24147, 34129, 6312, 78364, 67127, 34224, 60410, 4682, 40396, 1888, 34274, 34634, 64330, 78221, 46970, 34182, 69712, 33624, 34107, 4690, 34251, 59820, 64141, 76952, 77497, 26213, 20026, 27008, 24680, 2486, 2985, 8726, 7751, 8332, 19779, 182, 22070, 3777, 2525, 48065, 8878, 8831
Antidepressants	Amitriptyline Hydrochloride/ Chlordiazepoxide	21081, 18342, 11963, 14534
Antidepressants	Amitriptyline hydrochloride/ Perphenazine	3490, 595, 1453, 1208, 38827, 16323, 6894
Antidepressants	Amoxapine	3652, 4411, 17319, 3351, 21357, 24723, 15380, 14398, 55289
Antidepressants	Butriptyline Hydrochloride	12227, 32457, 18932
Antidepressants	Clomipramine	3195
Antidepressants	Clomipramine hydrochloride	30375, 26513, 7515, 3657, 8719, 7693, 7894, 3194, 34866, 68665, 41628, 62620, 43561, 3670, 34245, 41563, 45350, 65762, 8720, 64458, 3925, 45318, 41597, 53187, 78324, 65804, 53161, 38274, 78057, 8661
Antidepressants	Desipramine	7981, 7979
Antidepressants	Dosulepin hydrochloride	43024, 77130, 70838, 84, 23426, 34745, 34643, 31824, 44853, 29875, 33164, 34641, 76317, 34223, 50722, 71023, 70593, 74, 32121, 19186, 67728, 42734, 31826, 34525, 62681, 71059, 34058, 57926, 1940, 15632, 21820, 21819, 67990, 51758, 1169, 2320, 30376, 21157, 19168, 45737, 6054, 10948
Antidepressants	Doxepin hydrochloride	5190, 9558, 15975, 3842, 3554, 5073, 73363, 7059, 35258, 35493, 10413, 12129, 12125, 14519, 40777
Antidepressants	Fluphenazine hydrochloride/ Nortriptyline hydrochloride	2936, 7780
Antidepressants	Imipramine hydrochloride	1310, 41681, 34222, 67935, 71253, 70287, 32863, 34872, 1809, 34813, 34355, 41408, 8055, 42247, 33074, 2579, 56501, 7910, 4404
Antidepressants	Iprindole	27476, 27733, 24700, 31672
Antidepressants	Lofepramine	79397
Antidepressants	Lofepramine hydrochloride	58450, 2093, 41627, 114, 34046, 34950, 71067, 74586, 66100, 34578, 68657, 67742, 56703, 34672, 60591, 56229, 43534, 4218, 77717, 25444
Antidepressants	Mianserin hydrochloride	7468, 8144, 8585, 3083, 47363, 4329, 6255, 12368, 11956, 12192

Antidepressants	Nortriptyline hydrochloride	7677
		8640, 3183, 65237, 55970, 72626, 68228, 3903, 48216, 63276, 66201, 78224, 69317, 17183, 12549, 12353, 4118, 39145, 7678
Antidepressants	Nortriptyline hydrochloride/ Fluphenazine hydrochloride	8493, 14578, 20571
Antidepressants	Protriptyline hydrochloride	60929, 7755, 7816, 11187, 7756
Antidepressants	Trazodone hydrochloride	4194, 4003, 4874, 8174, 13621, 1730, 34580, 73639, 19181, 41709, 41710, 65152, 72291, 66749, 12710, 4020, 73419, 77915, 73636, 76480, 30983, 29857, 34470, 55137, 55138, 57226, 3355, 34003, 71031, 29339, 41609, 34421, 61842, 6442, 59931, 70521, 77474, 61657, 69355
Antidepressants	Trimipramine maleate	8928, 2532, 2531, 4310, 42228, 53808, 2039, 45226, 57978, 66493, 3196, 65445, 66919, 65213
Antidepressants	Viloxazine Hydrochloride	12309, 12111
Antidepressants	Citalopram hydrobromide	3861, 79784, 63953, 1712, 2408, 34498, 476, 34586, 64423, 32848, 49165, 42660, 52100, 59650, 53787, 71005, 33720, 52408, 34436, 45286, 75697, 52824, 59193, 63441, 34499, 60888, 41528, 56355, 34413, 54827, 34722, 67, 34356, 67097, 34871, 53394, 48026, 56009, 58476, 52607, 52354, 34415, 34970, 73417, 72373, 26016, 34966, 60568, 34822, 71848, 43519, 4770, 36746, 69571, 46977, 75075, 60839, 70790, 55033, 75702, 34603, 45223, 34466, 45304, 46926, 32546, 29756, 74753, 815, 513, 57936, 56292, 72124
Antidepressants	Escitalopram oxalate	74785, 648, 74858, 26056, 6360, 41062, 785, 603, 63916, 74993, 20152, 6218, 72773, 40726, 6405
Antidepressants	Fluoxetine hydrochloride	33071, 67431, 69941, 77881, 42499, 75645, 38890, 22, 19183, 71852, 45329, 60962, 75799, 67736, 45247, 75688, 34202, 34294, 69525, 59358, 66744, 34288, 42107, 62155, 19470, 45224, 67769, 34456, 34849, 67092, 45316, 33410, 60534, 60138, 2548, 34216, 42803, 60619, 73414, 30258, 36893, 68266, 69685, 74886, 67496, 79590, 67562, 75068, 78889, 4075, 75247, 67888, 34856, 62335, 14740, 67758, 77381, 418, 48220, 61335, 69542, 57532, 252, 75943, 4907, 37256, 33779, 29786
Antidepressants	Fluvoxamine maleate	12123, 2897, 2290, 48045, 44861, 43518, 2880
Antidepressants	Nefazodone hydrochloride	3391, 4297, 63827, 4554, 4011, 67757
Antidepressants	Paroxetine hydrochloride	35021, 76946, 59288, 67259, 527, 50, 34419, 32899, 73668, 40892, 34351, 55023, 33978, 1397, 34587, 40165, 64785, 78843, 68325, 35112, 66292, 74588, 841, 73589, 77650, 3601, 1575, 55537, 76772, 79383, 79381, 75054
Antidepressants	Sertraline	65771

Livingstone SJ, *et al. BMJMED* 2022; 1:e000316. doi: 10.1136/bmjmed-2022-000316

Antidepressants	Sertraline hydrochloride	4352, 77385, 1612, 727, 55146, 62950, 61503, 59600, 62692, 69726, 67928, 66560, 54933, 66413, 68756, 44944, 73962, 49519, 77607, 78278, 62819, 54826, 78626, 73759, 54081, 488, 32401, 58723, 42387, 45915, 62693, 69725, 63481, 58664, 67730, 69898, 55488, 75952, 62927, 75405, 7328, 77538, 77707
Antidepressants	Agomelatine	40494, 40295
Antidepressants	Duloxetine hydrochloride	74774, 7122, 13151, 62688, 63370, 65618, 65809, 66412, 70405, 70728, 73298, 74907, 79628, 6895, 14849, 51383, 63216, 63763, 64442, 65888, 65892, 66405, 68096, 69428, 69752, 69965, 72211, 73540, 73868, 74190, 78777, 76857
Antidepressants	Mirtazapine	6421, 43253, 64101, 43241, 66580, 61856, 43248, 43246, 68680, 55482, 58291, 77865, 65555, 43237, 48698, 54012, 6795, 43239, 53699, 66183, 59953, 46668, 66752, 43242, 54342, 54644, 74557, 43257, 16154, 53321, 61547, 47966, 68544, 6488, 43250, 53648, 48185, 68052, 69420, 76187, 59694, 742, 47945, 40160, 54792, 69005, 77488, 78654, 60538, 56209, 68933, 71543, 63403, 6481, 43235, 43236, 43256, 43247, 64139, 43234, 49820, 6854, 33337, 58625, 59954, 64223, 77377, 4726, 67272, 60370, 6846, 50892, 10083, 53543, 15268
Antidepressants	Nefazodone Hydrochloride	9534
Antidepressants	Reboxetine mesilate	15163, 2356
Antidepressants	Tryptophan	54747, 5611, 20504, 12221, 54686, 4422
Antidepressants	Venlafaxine hydrochloride	52516, 52074, 71806, 61236, 45664, 45959, 65738, 67271, 623, 6274, 67288, 77089, 9182, 74010, 5710, 51280, 65899, 74011, 75894, 1474, 76771, 43968, 43673, 41299, 48199, 41314, 41033, 59753, 60843, 40817, 40815, 39809, 39770, 57751, 52716, 40514, 40515, 70420, 70495, 69819, 70315, 50081, 59035, 49511, 58726, 74516, 58681, 55501, 2654, 70806, 60549, 71782, 43334, 39360, 50934, 62734, 65666, 40054, 58837, 45806, 301, 56662, 73667, 68050, 75525, 59923, 70353, 51361, 60895, 51699, 13237, 2617, 470, 71257, 59563, 68876, 43203, 39359, 1222, 60449, 73658, 66437, 56457, 63859, 53326, 63268, 40062, 40407, 45818, 40059, 44936, 44937, 71932, 70931, 40092, 67563, 40277, 76727, 75263, 40517, 42600, 40764, 40917, 40049, 78585, 40048, 75848, 55424
Antidepressants	Vortioxetine hydrobromide	67874, 69991, 69992, 65483, 66890, 65482
Antidepressants	Iproniazide	25945, 18290
Antidepressants	Isocarboxazid	41731, 12207, 12503
Antidepressants	Moclobemide	9206, 5832, 2883, 67305, 41747, 5187
Antidepressants	Phenelzine sulfate	3349, 4321
Antidepressants	Tranylcypromine sulfate	10787, 3783, 41654
Antidepressants	Trifluoperazine Hydrochloride/Tranylcypromine Sulphate	3356

Livingstone SJ, *et al. BMJMED* 2022; 1:e000316. doi: 10.1136/bmjmed-2022-000316

Antidepressants	Trifluoperazine Hydrochloride/ Tranylcypromine Sulphate	3955, 24890
Corticosteroids	Dexamethasone Sodium Phosphate]	28215, 37500, 14906, 61316, 53173, 19259, 47598, 61958, 56940, 35453, 10657, 13972, 26299, 13952, 26454, 31948, 34083, 4233
Corticosteroids	Hydrocortisone acetate	8108, 1893
Corticosteroids	Lidocaine Hydrochloride/ Methylprednisolone Acetate	925, 20157, 50253, 49076, 50734, 7405, 35156
Corticosteroids	Methylprednisolone	18042, 8261, 10683, 15555, 14172, 10552, 76923, 10684, 2130
Corticosteroids	Methylprednisolone acetate	48800, 48748, 48746, 14982, 71106, 27413, 33132, 35349, 35040, 35688, 1133, 5493
Corticosteroids	Methylprednisolone sodium succinate	18266, 13397, 12405, 18765, 14188, 25226, 25839, 23511, 21540
Corticosteroids	Triamcinolone acetonide	14962, 35578, 14335, 14958, 50216, 22047, 50026, 33131, 16583, 48406, 9368, 11123, 4488, 30244, 4125, 4123, 8864, 13981, 768, 37737, 3703, 16582
Corticosteroids	Triamcinolone hexacetonide	50854, 50853, 57856, 66867, 15016, 7992
Corticosteroids	Betamethasone	10864, 11149, 7286, 64235, 68306, 1971, 50225
Corticosteroids	Cortisone acetate	12398, 229, 53143, 7548, 53705, 18637, 12400, 10574, 23210
Corticosteroids	Deflazacort	22555, 29112, 20577, 41335, 9375, 78839, 17410, 3992
Corticosteroids	Dexamethasone	53207, 9994, 34801, 71926, 78335, 45234, 66724, 56443, 76339, 77085, 52396, 77849, 74156, 74157, 36055, 1280, 62909, 60120, 34880, 68182, 64747, 5157, 54793, 70611, 78214, 70893, 68489, 72537, 69572, 4779, 55401, 34915, 186, 74436, 56347, 68593, 73216, 21903
Corticosteroids	Hydrocortisone	75064, 74502, 75065, 76671, 3418, 65984, 64787, 66666, 38022, 75019, 51849, 51872, 64059, 54794, 4535, 66327, 57931, 75384, 77646, 51871, 75937, 52053, 75020, 53953, 63138, 14076, 51722, 51824, 75729, 74497, 71620, 38054, 10754, 6098, 13043, 77994, 58592, 59418
Corticosteroids	Hydrocortisone sodium phosphate	35172, 35175, 71905, 37638, 43355, 77821, 9574, 2615
Corticosteroids	Hydrocortisone sodium succinate	49707, 49498, 51167, 54715, 34166, 13350, 3754, 3651

Corticosteroids	Prednisolone	78546, 27962, 28859, 25272, 23512, 20095, 34914, 5913, 5490, 59283, 34631, 66645, 66015, 80110, 59229, 69568, 78129, 64007, 64008, 64009, 69686, 64128, 63172, 58234, 65626, 34109, 9727, 33691, 64416, 74239, 66914, 72421, 80050, 578, 34452, 34404, 73553, 58384, 63549, 28376, 2368, 38407, 61132, 75001, 34660, 51753, 34748, 56891, 34978, 59338, 557, 28375, 34461, 76020, 55480, 79930, 68497, 63066, 73294, 54434, 63082, 67076, 53313, 2704, 53336, 78144, 41745, 65020, 54118, 67507, 69811, 44, 31532, 32803, 66550, 67107, 73678, 58987, 34393, 59912, 45302, 75763, 33988, 33990, 95, 21417, 29333, 58000, 58369, 34781, 60421, 41515, 55024, 63791, 67559, 61162, 32835, 64221
Corticosteroids	Prednisolone sodium phosphate	1063, 47142, 955, 61689, 74493, 63214, 19141, 78789, 70603, 77760, 24224
Corticosteroids	Prednisolone Steaglate	31327, 3345
Corticosteroids	Prednisone	21833, 54432, 44803, 44802, 44380, 3557, 46711, 58061, 44723, 62656, 43544, 2949
Corticosteroids	Triamcinolone Acetonide	24014, 15617, 19908, 23111

Table S6: Missing data

	How missingness was handled in analysis	Women external validation cohort<br><br>N=2747409<br>No (%) missing data	Men external validation cohort<br><br>N=2684730<br>No (%) missing data	All patients original QFracture internal validation cohort<br>N=1583373<br>No (%) missing data
Age	Never missing	0	0	0
Sex	Never missing	0	0	0
Socioeconomic status	Excluded from cohort	0	0	0
Body mass index (BMI)	Imputed	932720 (34.0)	1233196 (45.9)	418478 (26.4)
Smoking status	Imputed	780226 (28.4)	963580 (35.9)	258144 (16.3)
Alcohol status	Imputed	698902 (25.4)	866622 (32.3)	461740 (29.2)
Ethnicity	Assumed to be white	1278931 (46.6)	1494450 (55.7)	855485 (54.0)
Conditions and prescribing variables	Assumed to be absent if no record	NA	NA	NA

Livingstone SJ, *et al. BMJMED* 2022; 1:e000316. doi: 10.1136/bmjmed-2022-000316

Table S7: Crude incidence of major osteoporotic fracture (MOF) over 10 years of follow-up

	Women			Men		
Age	Incident MOF	Total follow-up Years	Rate per 1000 person-year	Incident MOF	Total follow-up Years	Rate per 1000 person years
30-34	2,603	2741657	0.95 (0.91 to 0.99)	2,828	2784175	1.02 (0.98 to 1.05)
35-39	2,025	1870595	1.08 (1.04 to 1.13)	2,121	1927589	1.10 (1.05 to 1.15)
40-44	2,698	1833507	1.47 (1.42 to 1.53)	2,222	1917796	1.16 (1.11 to 1.21)
45-49	3,633	1595805	2.28 (2.20 to 2.35)	2,239	1681808	1.33 (1.28 to 1.39)
50-54	5,292	1449369	3.65 (3.55 to 3.75)	2,248	1497499	1.50 (1.44 to 1.56)
55-59	7,422	1490080	4.98 (4.87 to 5.10)	2,644	1505675	1.76 (1.69 to 1.82)
60-64	7,762	1210157	6.41 (6.27 to 6.56)	2,743	1191801	2.30 (2.22 to 2.39)
65-69	9,455	1024227	9.23 (9.05 to 9.42)	2,859	960815	2.98 (2.87 to 3.09)
70-74	11,757	861260	13.65 (13.41 to 13.90)	3,456	748844	4.62 (4.46 to 4.77)
75-80	14,148	688855	20.54 (20.21 to 20.88)	4,068	516507	7.88 (7.64 to 8.12)
80-84	14,653	508415	28.82 (28.36 to 29.28)	3,891	304005	12.80 (12.41 to 13.20)
85-90	9,017	237728	37.93 (37.17 to 38.71)	2,080	107018	19.44 (18.63 to 20.28)
90-99	5,133	112888	45.47 (44.27 to 46.70)	922	36093	25.55 (23.97 to 27.22)
Total	95,598	15624543	6.12 (6.08 to 6.16)	34,321	15179623	2.26 (2.24 to 2.29)

Table S8: Crude incidence of hip fracture over 10 years of follow-up

	Women			Men		
Age	Incident hip fractures	Total Follow-up Years	Rate per 1000 person-year	Incident hip fractures	Total Follow-up Years	Rate per 1000 person years
30-34	93	2750441	0.03 (0.03 to 0.04)	214	2793615	0.08 (0.07 to 0.09)
35-39	109	1878222	0.06 (0.05 to 0.07)	223	1935329	0.12 (0.10 to 0.13)
40-44	183	1842965	0.10 (0.09 to 0.11)	307	1925573	0.16 (0.14 to 0.18)
45-49	374	1607632	0.23 (0.21 to 0.26)	377	1689307	0.22 (0.20 to 0.25)
50-54	599	1467062	0.41 (0.38 to 0.44)	442	1504825	0.29 (0.27 to 0.32)
55-59	1,149	1515268	0.76 (0.72 to 0.80)	701	1513119	0.46 (0.43 to 0.50)
60-64	1,554	1234523	1.26 (1.20 to 1.32)	948	1197990	0.79 (0.74 to 0.84)
65-69	2,614	1051678	2.49 (2.39 to 2.58)	1,217	966352	1.26 (1.19 to 1.33)
70-74	4,460	889669	5.01 (4.87 to 5.16)	1,709	754325	2.27 (2.16 to 2.38)
75-80	6,905	715572	9.65 (9.43 to 9.88)	2,432	521184	4.67 (4.48 to 4.86)
80-84	8,752	527816	16.58 (16.24 to 16.93)	2,640	307196	8.59 (8.27 to 8.93)
85-90	5,968	246247	24.24 (23.64 to 24.85)	1,469	108226	13.57 (12.90 to 14.28)
90-99	3,640	115681	31.47 (30.48 to 32.49)	700	36423	19.22 (17.86 to 20.68)
Total	36,400	15842775	2.30 (2.27 to 2.32)	13,379	15253462	0.88 (0.86 to 0.89)

Livingstone SJ, *et al. BMJMED* 2022; 1:e000316. doi: 10.1136/bmjmed-2022-000316

Table S9: Comparison of major osteoporotic fracture (MOF) incidence in this study and previous external validation study[a 3]

	Women		Men	
Age	MOF rate/1000 person-years (this study)	MOF rate/1000 person-years (previous external validation)	MOF rate/1000 person-years (this study)	MOF rate/1000 person-years (previous external validation)
30-34	0.95 (0.91 to 0.99)	0.42 (0.38 to 0.46)	1.02 (0.98 to 1.05)	0.45 (0.41 to 0.50)
35-39	1.08 (1.04 to 1.13)	0.44 (0.40 to 0.49)	1.10 (1.05 to 1.15)	0.45 (0.41 to 0.49)
40-44	1.47 (1.42 to 1.53)	0.60 (0.55 to 0.66)	1.16 (1.11 to 1.21)	0.44 (0.39 to 0.49)
45-49	2.28 (2.20 to 2.35)	0.96 (0.89 to 1.04)	1.33 (1.28 to 1.39)	0.55 (0.50 to 0.61)
50-54	3.65 (3.55 to 3.75)	1.57 (1.48 to 1.66)	1.50 (1.44 to 1.56)	0.67 (0.61 to 0.73)
55-59	4.98 (4.87 to 5.10)	2.22 (2.10 to 2.34)	1.76 (1.69 to 1.82)	0.74 (0.67 to 0.81)
60-64	6.41 (6.27 to 6.56)	3.54 (3.37 to 3.70)	2.30 (2.22 to 2.39)	0.98 (0.89 to 1.07)
65-69	9.23 (9.05 to 9.42)	5.15 (4.94 to 5.36)	2.98 (2.87 to 3.09)	1.42 (1.31 to 1.54)
70-74	13.65 (13.41 to 13.90)	8.07 (7.79 to 8.36)	4.62 (4.46 to 4.77)	2.69 (2.52 to 2.88)
75-80	20.54 (20.21 to 20.88)	11.96 (11.57 to 12.35)	7.88 (7.64 to 8.12)	4.03 (3.77 to 4.31)
80-84	28.82 (28.36 to 29.28)	17.70 (17.14 to 18.28)	12.80 (12.41 to 13.20)	7.01 (6.54 to 7.51)
85-89 [b]	37.93 (37.17 to 38.71)	-	19.44 (18.63 to 20.28)	-
90-99 [b]	45.47 (44.27 to 46.70)	-	25.55 (23.97 to 27.22)	-
Total	6.12 (6.08 to 6.16)	2.93 (2.89 to 2.98)	2.26 (2.24 to 2.29)	0.98 (0.95 to 1.00)

a. QFracture derivation papers do not report incidence by age, so the external validation study data is the comparison. As with QFracture derivation and internal validation, the external validation study ascertained fractures using GP electronic health record data and mortality registration data, whereas this study also used fractures recorded at hospital discharge

b. Previous external validation study maximum age is 85; in this study maximum age is 99

Livingstone SJ, *et al. BMJMED* 2022; 1:e000316. doi: 10.1136/bmjmed-2022-000316

Table S10: Comparison of hip fracture incidence in this study and previous external validation study[a 3]

	Women		Men	
Age	Hip fracture rate/1000 person-years (this study)	Hip fracture rate/1000 person-years (previous external validation)	Hip fracture rate/1000 person-years (this study)	Hip fracture rate/1000 person-years (previous external validation)
30-34	0.03 (0.03 to 0.04)	0.03 (0.02 to 0.05)	0.08 (0.07 to 0.09)	0.05 (0.04 to 0.06)
35-39	0.06 (0.05 to 0.07)	0.04 (0.03 to 0.06)	0.12 (0.10 to 0.13)	0.08 (0.07 to 0.10)
40-44	0.10 (0.09 to 0.11)	0.08 (0.06 to 0.10)	0.16 (0.14 to 0.18)	0.09 (0.07 to 0.12)
45-49	0.23 (0.21 to 0.26)	0.18 (0.15 to 0.21)	0.22 (0.20 to 0.25)	0.15 (0.13 to 0.18)
50-54	0.41 (0.38 to 0.44)	0.31 (0.27 to 0.35)	0.29 (0.27 to 0.32)	0.22 (0.19 to 0.26)
55-59	0.76 (0.72 to 0.80)	0.56 (0.50 to 0.62)	0.46 (0.43 to 0.50)	0.31 (0.27 to 0.36)
60-64	1.26 (1.20 to 1.32)	1.01 (0.93 to 1.10)	0.79 (0.74 to 0.84)	0.43 (0.37 to 0.49)
65-69	2.49 (2.39 to 2.58)	1.97 (1.85 to 2.10)	1.26 (1.19 to 1.33)	0.79 (0.70 to 0.87)
70-74	5.01 (4.87 to 5.16)	3.97 (3.78 to 4.17)	2.27 (2.16 to 2.38)	1.67 (1.54 to 1.82)
75-80	9.65 (9.43 to 9.88)	7.03 (6.75 to 7.32)	4.67 (4.48 to 4.86)	2.84 (2.62 to 3.08)
80-84	16.58 (16.24 to 16.93)	12.47 (12.02 to 12.94)	8.59 (8.27 to 8.93)	5.42 (5.01 to 5.86)
85-90 [b]	24.24 (23.64 to 24.85)		13.57 (12.90 to 14.28)	
90-99 [b]	31.47 (30.48 to 32.49)		19.22 (17.86 to 20.68)	
Total	2.30 (2.27 to 2.32)	1.37 (1.35 to 1.40)	0.88 (0.86 to 0.89)	0.47 (0.46 to 0.49)

a. QFracture derivation papers do not report incidence by age, so the external validation study data is the comparison. As with QFracture derivation and internal validation, the external validation study ascertained fractures using GP electronic health record data and mortality registration data, whereas this study also used fractures recorded at hospital discharge

b. Previous external validation study maximum age is 85; in this study maximum age is 99; reported previous external validation rate is therefore for age 80-85

Table S11: Crude incidence of major osteoporotic fracture (MOF) over 10 years of follow-up (with ascertainment restricted to GP and mortality data)

	Women			Men		
Age	Incident MOF	Total follow-up Years	Rate per 1000 person-year	Incident MOF	Total follow-up Years	Rate per 1000 person years
30-34	2,316	2742607	0.84 (0.81 to 0.88)	2,447	2785431	0.88 (0.84 to 0.91)
35-39	1,788	1871469	0.96 (0.91 to 1.00)	1,848	1928588	0.96 (0.92 to 1.00)
40-44	2,387	1834615	1.30 (1.25 to 1.35)	1,897	1918981	0.99 (0.95 to 1.03)
45-49	3,212	1597368	2.01 (1.94 to 2.08)	1,914	1682894	1.14 (1.09 to 1.19)
50-54	4,747	1451234	3.27 (3.18 to 3.37)	1,902	1498727	1.27 (1.21 to 1.33)
55-59	6,569	1493186	4.40 (4.29 to 4.51)	2,160	1507164	1.43 (1.37 to 1.49)
60-64	6,829	1213292	5.63 (5.50 to 5.76)	2,221	1193239	1.86 (1.79 to 1.94)
65-69	8,116	1028487	7.89 (7.72 to 8.06)	2,293	962282	2.38 (2.29 to 2.48)
70-74	9,965	866524	11.50 (11.28 to 11.73)	2,752	750538	3.67 (3.53 to 3.81)
75-80	11,693	695373	16.82 (16.52 to 17.12)	3,247	518140	6.27 (6.06 to 6.49)
80-84	11,873	514580	23.07 (22.67 to 23.49)	3,041	305425	9.96 (9.61 to 10.31)
85-89	7,175	241340	29.73 (29.06 to 30.41)	1,576	107622	14.64 (13.94 to 15.38)
90-99	4,126	114095	36.16 (35.09 to 37.26)	718	36261	19.80 (18.42 to 21.29)
Total	80,796	15664170	5.16 (5.12 to 5.19)	28,016	15195293	1.84 (1.82 to 1.87)

Table S12: Comparison of major osteoporotic fracture incidence in this study with complete fracture ascertainment (GP, mortality and hospital admission data), previous external validation study, and in this study using ascertainment to match previous study (GP and mortality data only) [3]

	Women			Men		
Age	MOF rate/1000 person-years (this study)[a]	MOF rate/1000 person-years (previously published external validation)[b]	MOF rate/1000 person-years (this study matched ascertainment)[b]	MOF rate/1000 person-years (this study)[a]	MOF rate/1000 person-years (previously published external validation)[b]	MOF rate/1000 person-years (this study matched ascertainment)[b]
30-34	0.95 (0.91 to 0.99)	0.03 (0.02 to 0.05)	0.84 (0.81 to 0.88)	1.02 (0.98 to 1.05)	0.05 (0.04 to 0.06)	0.88 (0.84 to 0.91)
35-39	1.08 (1.04 to 1.13)	0.04 (0.03 to 0.06)	0.96 (0.91 to 1.00)	1.10 (1.05 to 1.15)	0.08 (0.07 to 0.10)	0.96 (0.92 to 1.00)
40-44	1.47 (1.42 to 1.53)	0.08 (0.06 to 0.10)	1.30 (1.25 to 1.35)	1.16 (1.11 to 1.21)	0.09 (0.07 to 0.12)	0.99 (0.95 to 1.03)
45-49	2.28 (2.20 to 2.35)	0.18 (0.15 to 0.21)	2.01 (1.94 to 2.08)	1.33 (1.28 to 1.39)	0.15 (0.13 to 0.18)	1.14 (1.09 to 1.19)
50-54	3.65 (3.55 to 3.75)	0.31 (0.27 to 0.35)	3.27 (3.18 to 3.37)	1.50 (1.44 to 1.56)	0.22 (0.19 to 0.26)	1.27 (1.21 to 1.33)
55-59	4.98 (4.87 to 5.10)	0.56 (0.50 to 0.62)	4.40 (4.29 to 4.51)	1.76 (1.69 to 1.82)	0.31 (0.27 to 0.36)	1.43 (1.37 to 1.49)
60-64	6.41 (6.27 to 6.56)	1.01 (0.93 to 1.10)	5.63 (5.50 to 5.76)	2.30 (2.22 to 2.39)	0.43 (0.37 to 0.49)	1.86 (1.79 to 1.94)
65-69	9.23 (9.05 to 9.42)	1.97 (1.85 to 2.10)	7.89 (7.72 to 8.06)	2.98 (2.87 to 3.09)	0.79 (0.70 to 0.87)	2.38 (2.29 to 2.48)
70-74	13.65 (13.41 to 13.90)	3.97 (3.78 to 4.17)	11.50 (11.28 to 11.73)	4.62 (4.46 to 4.77)	1.67 (1.54 to 1.82)	3.67 (3.53 to 3.81)
75-80	20.54 (20.21 to 20.88)	7.03 (6.75 to 7.32)	16.82 (16.52 to 17.12)	7.88 (7.64 to 8.12)	2.84 (2.62 to 3.08)	6.27 (6.06 to 6.49)
80-84	28.82 (28.36 to 29.28)	12.47 (12.02 to 12.94)	23.07 (22.67 to 23.49)	12.80 (12.41 to 13.20)	5.42 (5.01 to 5.86	9.96 (9.61 to 10.31)
85-90 [c]	37.93 (37.17 to 38.71)	-	29.73 (29.06 to 30.41)	19.44 (18.63 to 20.28)	-	14.64 (13.94 to 15.38)
90-99 [c]	45.47 (44.27 to 46.70)	-	36.16 (35.09 to 37.26)	25.55 (23.97 to 27.22)	-	19.80 (18.42 to 21.29)
Total	6.12 (6.08 to 6.16)	1.37 (1.35 to 1.40)	5.16 (5.12 to 5.19)	2.26 (2.24 to 2.29)	0.47 (0.46 to 0.49)	1.84 (1.82 to 1.87)

a. Fractures ascertained using GP electronic health record data, mortality registration data, and fractures recorded at hospital discharge

b. Fractures ascertained using GP electronic health record data and mortality registration data (but NOT hospital discharge data)

c. Previous external validation study maximum age is 85; in this study maximum age is 99; reported previous external validation rate is therefore for age 80-85

Table S13: Crude incidence of hip fracture over 10 years of follow-up (with ascertainment restricted to GP and mortality data)

	Women			Men		
Age	Incident hip fracture	Total follow-up Years	Rate per 1000 person-year	Incident hip fracture	Total follow-up Years	Rate per 1000 person years
30-34	80	2750475	0.03 (0.02 to 0.04)	178	2793683	0.06 (0.06 to 0.07)
35-39	92	1878252	0.05 (0.04 to 0.06)	192	1935416	0.10 (0.09 to 0.11)
40-44	158	1843032	0.09 (0.07 to 0.10)	254	1925741	0.13 (0.12 to 0.15)
45-49	325	1607799	0.20 (0.18 to 0.23)	322	1689484	0.19 (0.17 to 0.21)
50-54	524	1467273	0.36 (0.33 to 0.39)	369	1505015	0.25 (0.22 to 0.27)
55-59	975	1515801	0.64 (0.60 to 0.68)	571	1513458	0.38 (0.35 to 0.41)
60-64	1,325	1235112	1.07 (1.02 to 1.13)	792	1198354	0.66 (0.62 to 0.71)
65-69	2,194	1052811	2.08 (2.00 to 2.17)	993	966812	1.03 (0.97 to 1.09)
70-74	3,644	891687	4.09 (3.96 to 4.22)	1,388	754978	1.84 (1.74 to 1.94)
75-80	5,570	718657	7.75 (7.55 to 7.96)	1,970	521983	3.77 (3.61 to 3.94)
80-84	6,992	531298	13.16 (12.86 to 13.47)	2,100	307978	6.82 (6.53 to 7.12)
85-90	4,700	248552	18.91 (18.38 to 19.45)	1,137	108580	10.47 (9.88 to 11.09)
90-99	2,910	116419	25.00 (24.11 to 25.91)	558	36527	15.28 (14.07 to 16.59)
Total	29,489	15857168	1.86 (1.84 to 1.88)	10,824	15258010	0.71 (0.70 to 0.72)

Table S14: Comparison of hip fracture incidence in this study with complete fracture ascertainment (GP, mortality and hospital admission data), previous external validation study, and in this study using ascertainment to match previous study (GP and mortality data only)[a 3]

	Women			Men		
Age	Hip fracture rate/1000 person-years (this study)[a]	Hip fracture rate/1000 person-years (previously published external validation)[b]	Hip fracture rate/1000 person-years (this study matched ascertainment)[b]	Hip fracture rate/1000 person-years (this study)[a]	Hip fracture rate/1000 person-years (previously published external validation)[b]	Hip fracture rate/1000 person-years (this study matched ascertainment)[b]
30-34	0.03 (0.03 to 0.04)	0.03 (0.02 to 0.05)	0.03 (0.02 to 0.04)	0.08 (0.07 to 0.09)	0.05 (0.04 to 0.06)	0.06 (0.06 to 0.07)
35-39	0.06 (0.05 to 0.07)	0.04 (0.03 to 0.06)	0.05 (0.04 to 0.06)	0.12 (0.10 to 0.13)	0.08 (0.07 to 0.10)	0.10 (0.09 to 0.11)
40-44	0.10 (0.09 to 0.11)	0.08 (0.06 to 0.10)	0.09 (0.07 to 0.10)	0.16 (0.14 to 0.18)	0.09 (0.07 to 0.12)	0.13 (0.12 to 0.15)
45-49	0.23 (0.21 to 0.26)	0.18 (0.15 to 0.21)	0.20 (0.18 to 0.23)	0.22 (0.20 to 0.25)	0.15 (0.13 to 0.18)	0.19 (0.17 to 0.21)
50-54	0.41 (0.38 to 0.44)	0.31 (0.27 to 0.35)	0.36 (0.33 to 0.39)	0.29 (0.27 to 0.32)	0.22 (0.19 to 0.26)	0.25 (0.22 to 0.27)
55-59	0.76 (0.72 to 0.80)	0.56 (0.50 to 0.62)	0.64 (0.60 to 0.68)	0.46 (0.43 to 0.50)	0.31 (0.27 to 0.36)	0.38 (0.35 to 0.41)
60-64	1.26 (1.20 to 1.32)	1.01 (0.93 to 1.10)	1.07 (1.02 to 1.13)	0.79 (0.74 to 0.84)	0.43 (0.37 to 0.49)	0.66 (0.62 to 0.71)
65-69	2.49 (2.39 to 2.58)	1.97 (1.85 to 2.10)	2.08 (2.00 to 2.17)	1.26 (1.19 to 1.33)	0.79 (0.70 to 0.87)	1.03 (0.97 to 1.09)
70-74	5.01 (4.87 to 5.16)	3.97 (3.78 to 4.17)	4.09 (3.96 to 4.22)	2.27 (2.16 to 2.38)	1.67 (1.54 to 1.82)	1.84 (1.74 to 1.94)
75-80	9.65 (9.43 to 9.88)	7.03 (6.75 to 7.32)	7.75 (7.55 to 7.96)	4.67 (4.48 to 4.86)	2.84 (2.62 to 3.08)	3.77 (3.61 to 3.94)
80-84	16.58 (16.24 to 16.93)	12.47 (12.02 to 12.94)	13.16 (12.86 to 13.47)	8.59 (8.27 to 8.93)	5.42 (5.01 to 5.86	6.82 (6.53 to 7.12)
85-90[c]	24.24 (23.64 to 24.85)	-	18.91 (18.38 to 19.45)	13.57 (12.90 to 14.28)	-	10.47 (9.88 to 11.09)
90-99[c]	31.47 (30.48 to 32.49)	-	25.00 (24.11 to 25.91)	19.22 (17.86 to 20.68)	-	15.28 (14.07 to 16.59)
Total	2.30 (2.27 to 2.32)	1.37 (1.35 to 1.40)	1.86 (1.84 to 1.88)	0.88 (0.86 to 0.89)	0.47 (0.46 to 0.49)	0.71 (0.70 to 0.72)

a. Fractures ascertained using GP electronic health record data, mortality registration data, and fractures recorded at hospital discharge

b. Fractures ascertained using GP electronic health record data and mortality registration data (but NOT hospital discharge data)

c. Previous external validation study maximum age is 85; in this study maximum age is 99; reported previous external validation rate is therefore for age 80-85

Livingstone SJ, *et al. BMJMED* 2022; 1:e000316. doi: 10.1136/bmjmed-2022-000316

Figure S1: Comparison of fracture incidence in this study (using GP, mortality and hospital admission data), previous external validation (using GP and ONS data but maximum age 85)[3] and this study matched to previous external validation ascertainment (using GP and ONS data)*

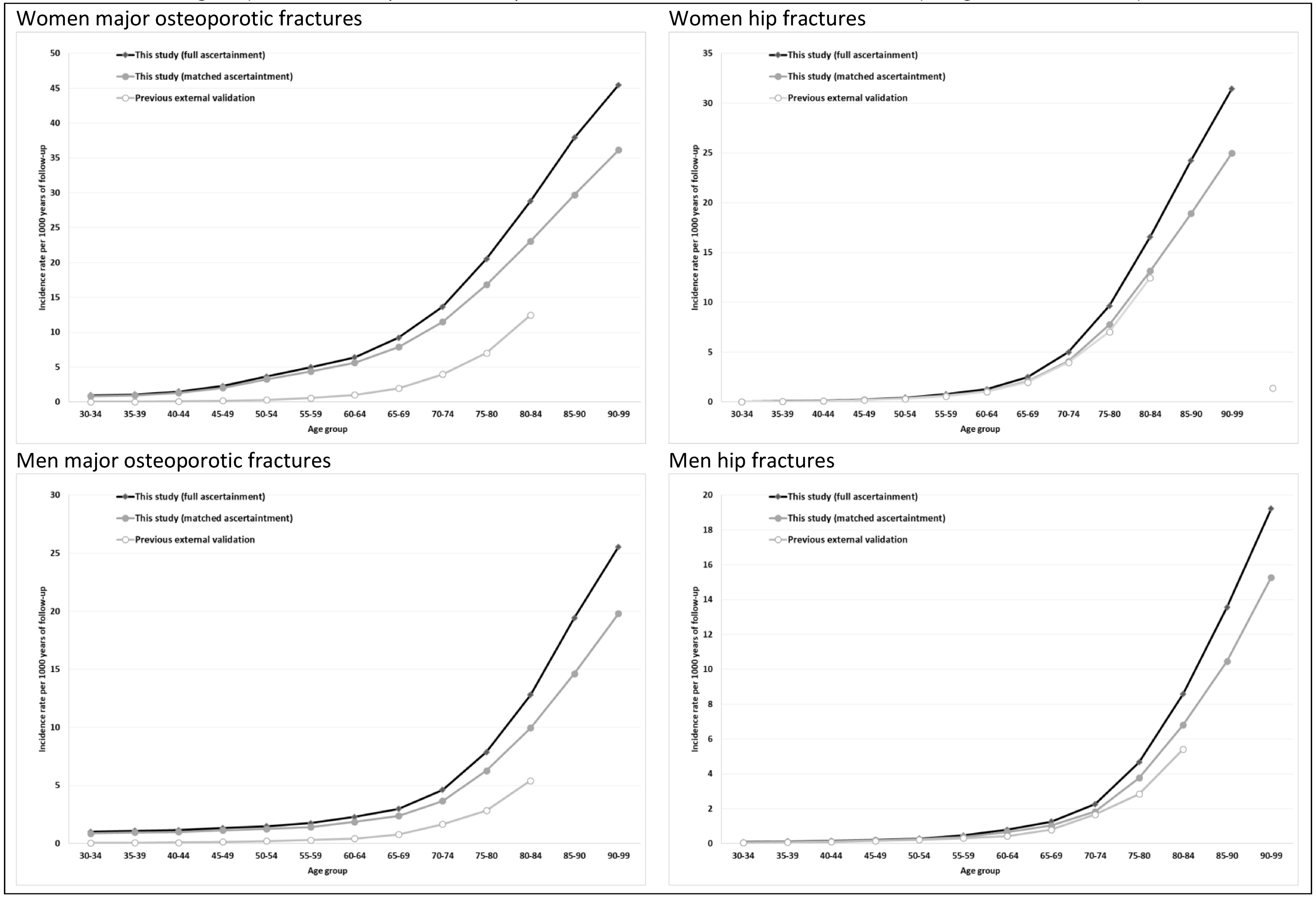

* The previous external validation[3] is of the first version of the QFracture tool[4] but the derivation paper for the second version[5] being evaluated in this study does not report fracture incidence by age. Key differences are that the external validation study only includes patients to age 84 years (vs to 99 years in this study), and excludes people with prior major osteoporotic fracture (who are included in this study, since prior MOF is a predictor)

Table S15: Crude incidence of non-fracture death over 10 years of follow-up

	Women			Men		
Age	Incident non-fracture death	Total follow-up Years	Rate per 1000 person-year	Incident non-fracture death	Total follow-up Years	Rate per 1000 person years
30-34	1,348	2741657	0.49 (0.47 to 0.52)	2,346	2784175	0.84 (0.81 to 0.88)
35-39	1,677	1870595	0.90 (0.85 to 0.94)	2,411	1927589	1.25 (1.20 to 1.30)
40-44	2,534	1833507	1.38 (1.33 to 1.44)	3,605	1917796	1.88 (1.82 to 1.94)
45-49	3,714	1595805	2.33 (2.25 to 2.40)	5,094	1681808	3.03 (2.95 to 3.11)
50-54	4,991	1449369	3.44 (3.35 to 3.54)	7,398	1497499	4.94 (4.83 to 5.05)
55-59	7,996	1490080	5.37 (5.25 to 5.48)	12,167	1505675	8.08 (7.94 to 8.23)
60-64	10,378	1210157	8.58 (8.41 to 8.74)	15,427	1191801	12.94 (12.74 to 13.15)
65-69	14,216	1024227	13.88 (13.65 to 14.11)	20,779	960815	21.63 (21.34 to 21.92)
70-74	19,734	861260	22.91 (22.60 to 23.23)	26,842	748844	35.84 (35.43 to 36.27)
75-80	27,874	688855	40.46 (40.00 to 40.93)	31,087	516507	60.19 (59.54 to 60.84)
80-84	36,030	508415	70.87 (70.17 to 71.58)	30,228	304005	99.43 (98.37 to 100.50)
85-89	29,415	237728	123.73 (122.42 to 125.06)	16,832	107018	157.28 (155.11 to 159.48)
90-99	23,799	112888	210.82 (208.45 to 213.21)	8,865	36093	245.62 (241.20 to 250.09)
Total	183,706	15624543	11.76 (11.70 to 11.81)	183,081	15179623	12.06 (12.01 to 12.12)

Table S16: Comparison of major osteoporotic fracture (MOF), hip fracture and non-fracture death incidence (rate per 1000 person/years [95%CI])

	Women			Men		
Age	Major osteoporotic fracture	Hip fracture	Non-fracture death	Major osteoporotic fracture	Hip fracture	Non-fracture death
30-34	0.95 (0.91 to 0.99)	0.03 (0.03 to 0.04)	0.49 (0.47 to 0.52)	1.02 (0.98 to 1.05)	0.08 (0.07 to 0.09)	0.84 (0.81 to 0.88)
35-39	1.08 (1.04 to 1.13)	0.06 (0.05 to 0.07)	0.90 (0.85 to 0.94)	1.10 (1.05 to 1.15)	0.12 (0.10 to 0.13)	1.25 (1.20 to 1.30)
40-44	1.47 (1.42 to 1.53)	0.10 (0.09 to 0.11)	1.38 (1.33 to 1.44)	1.16 (1.11 to 1.21)	0.16 (0.14 to 0.18)	1.88 (1.82 to 1.94)
45-49	2.28 (2.20 to 2.35)	0.23 (0.21 to 0.26)	2.33 (2.25 to 2.40)	1.33 (1.28 to 1.39)	0.22 (0.20 to 0.25)	3.03 (2.95 to 3.11)
50-54	3.65 (3.55 to 3.75)	0.41 (0.38 to 0.44)	3.44 (3.35 to 3.54)	1.50 (1.44 to 1.56)	0.29 (0.27 to 0.32)	4.94 (4.83 to 5.05)
55-59	4.98 (4.87 to 5.10)	0.76 (0.72 to 0.80)	5.37 (5.25 to 5.48)	1.76 (1.69 to 1.82)	0.46 (0.43 to 0.50)	8.08 (7.94 to 8.23)
60-64	6.41 (6.27 to 6.56)	1.26 (1.20 to 1.32)	8.58 (8.41 to 8.74)	2.30 (2.22 to 2.39)	0.79 (0.74 to 0.84)	12.94 (12.74 to 13.15)
65-69	9.23 (9.05 to 9.42)	2.49 (2.39 to 2.58)	13.88 (13.65 to 14.11)	2.98 (2.87 to 3.09)	1.26 (1.19 to 1.33)	21.63 (21.34 to 21.92)
70-74	13.65 (13.41 to 13.90)	5.01 (4.87 to 5.16)	22.91 (22.60 to 23.23)	4.62 (4.46 to 4.77)	2.27 (2.16 to 2.38)	35.84 (35.43 to 36.27)
75-80	20.54 (20.21 to 20.88)	9.65 (9.43 to 9.88)	40.46 (40.00 to 40.93)	7.88 (7.64 to 8.12)	4.67 (4.48 to 4.86)	60.19 (59.54 to 60.84)
80-84	28.82 (28.36 to 29.28)	16.58 (16.24 to 16.93)	70.87 (70.17 to 71.58)	12.80 (12.41 to 13.20)	8.59 (8.27 to 8.93)	99.43 (98.37 to 100.50)
85-89	37.93 (37.17 to 38.71)	24.24 (23.64 to 24.85)	123.73 (122.42 to 125.06)	19.44 (18.63 to 20.28)	13.57 (12.90 to 14.28)	157.28 (155.11 to 159.48)
90-99	45.47 (44.27 to 46.70)	31.47 (30.48 to 32.49)	210.82 (208.45 to 213.21)	25.55 (23.97 to 27.22)	19.22 (17.86 to 20.68)	245.62 (241.20 to 250.09)
Total	6.12 (6.08 to 6.16)	2.30 (2.27 to 2.32)	11.76 (11.70 to 11.81)	2.26 (2.24 to 2.29)	0.88 (0.86 to 0.89)	12.06 (12.01 to 12.12)

Livingstone SJ, *et al. BMJMED* 2022; 1:e000316. doi: 10.1136/bmjmed-2022-000316

Figure S2: Calibration for major osteoporotic fracture in women by agegroup without accounting for competing risks (left hand) and accounting for competing risks (right hand).

Women (not accounting for competing risks)

1a: Aged 30-64

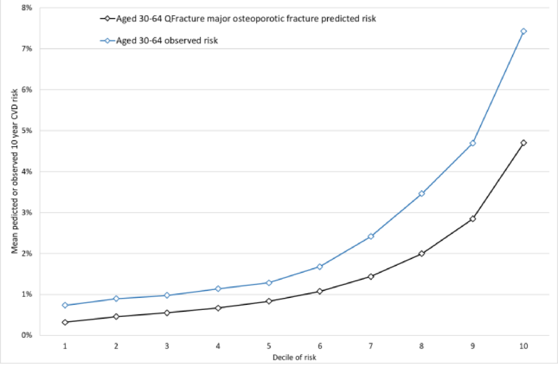

1c: Aged 65-74

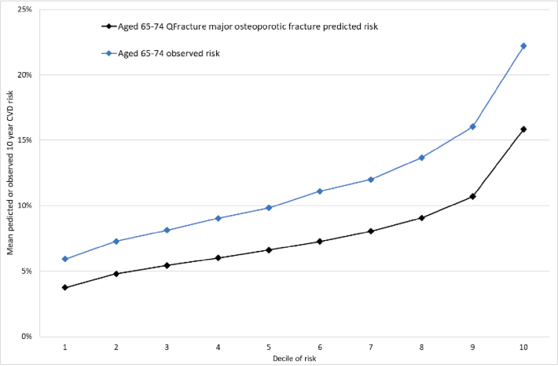

1e: Aged 75-84

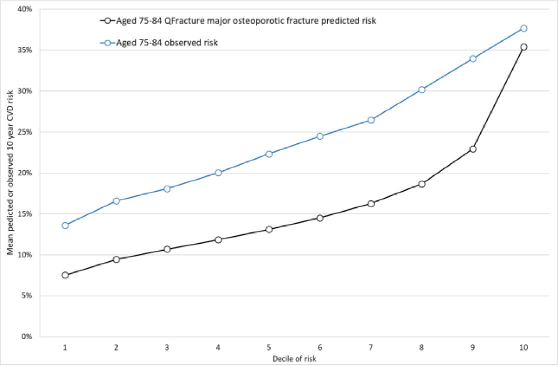

1g: Aged 85-99

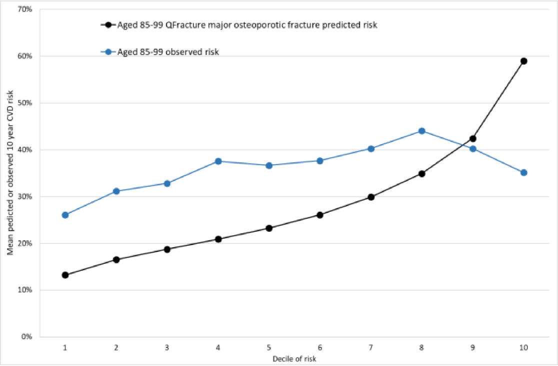

Women (accounting for competing risks)

1b: Aged 30-64

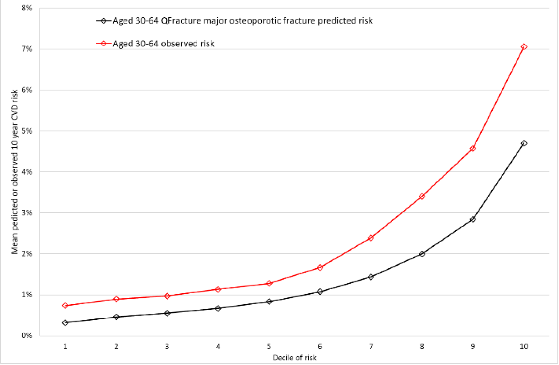

1d: Aged 65-74

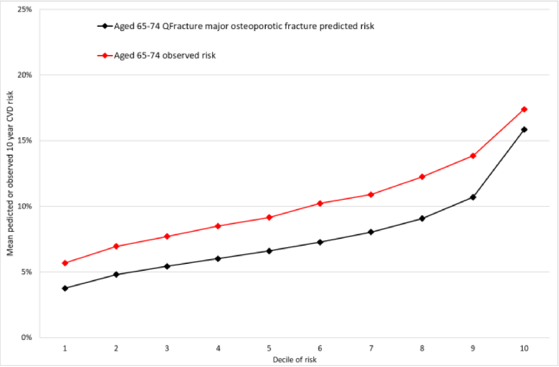

1f: Aged 75-84

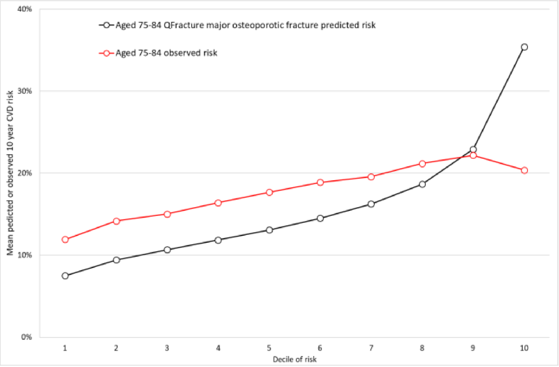

1h: Aged 85-99

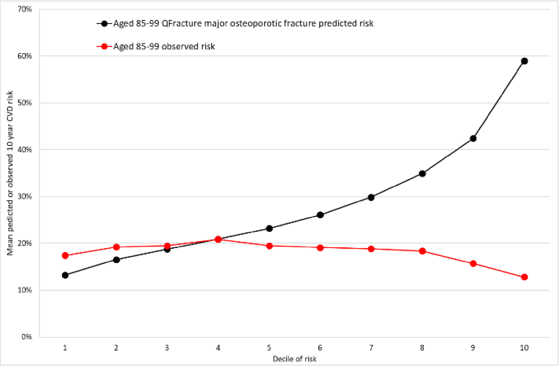

* Observed risk is based on the Kaplan-Meier estimator which does not account for competing mortality risk.
# Observed risk is based on the Aalen-Johansen estimator which accounts for competing mortality risk
Coloured line (observed risk) above matching black line (predicted risk) indicates under-prediction; below indicates over-prediction

Livingstone SJ, *et al. BMJMED* 2022; 1:e000316. doi: 10.1136/bmjmed-2022-000316

Figure S3: Calibration for major osteoporotic fracture in men by agegroup without accounting for competing risks (left hand) and accounting for competing risks (right hand)

Men (not accounting for competing risks)

1a: Aged 30-64

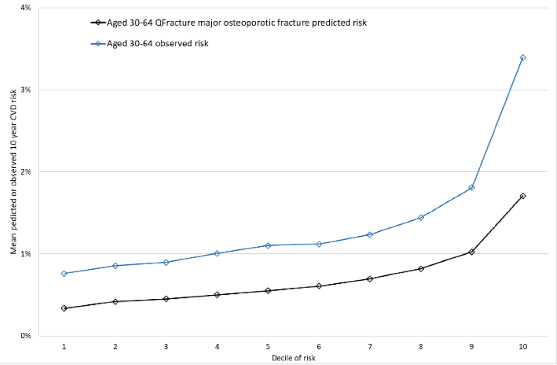

Men (accounting for competing risks)

1b: Aged 30-64

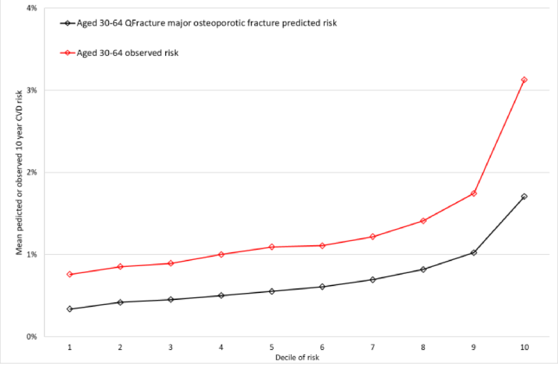

1c: Aged 65-74

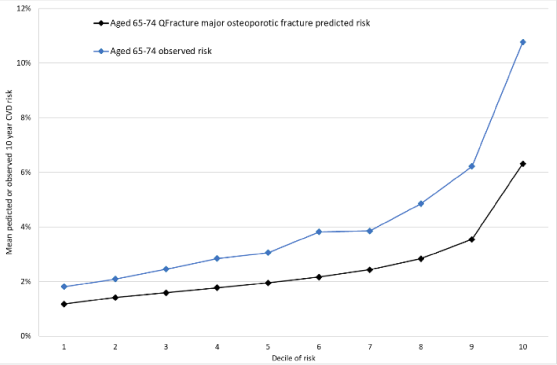

1d: Aged 65-74

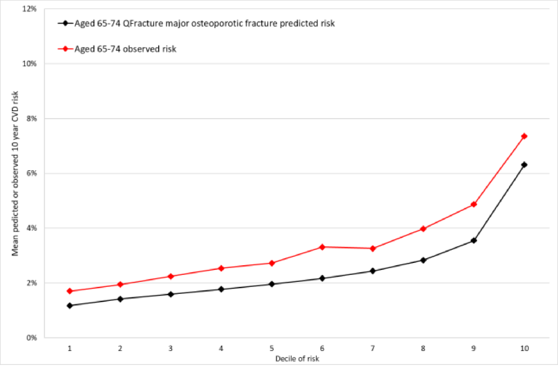

1e: Aged 75-84

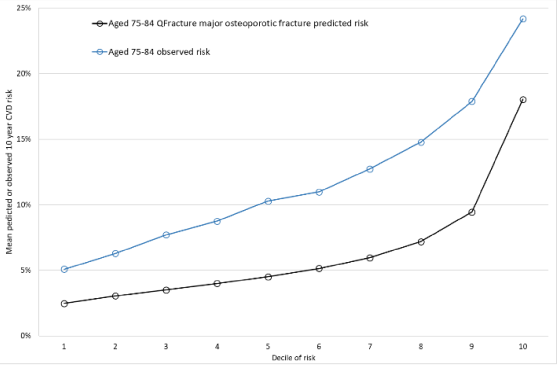

1f: Aged 75-84

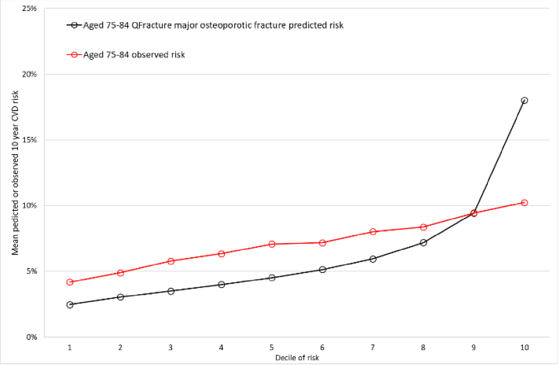

1g: Aged 85-99

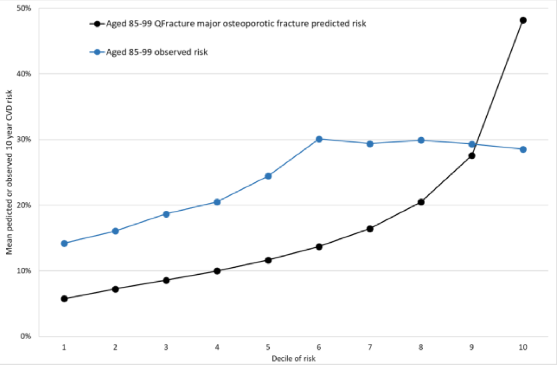

1h: Aged 85-99

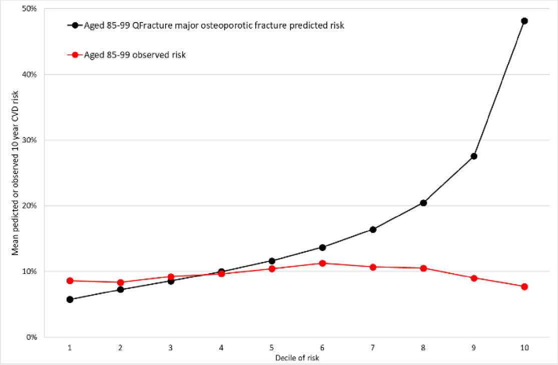

* Observed risk is based on the Kaplan-Meier estimator which does not account for competing mortality risk.
# Observed risk is based on the Aalen-Johansen estimator which accounts for competing mortality risk
Coloured line (observed risk) above matching black line (predicted risk) indicates under-prediction; below indicates over-prediction risk

32

Figure S4: Calibration for major osteoporotic fracture in women by Charlson Score without accounting for competing risks (left hand) and accounting for competing risks (right hand)

Women (not accounting for competing risks)

1a: mCCI=0

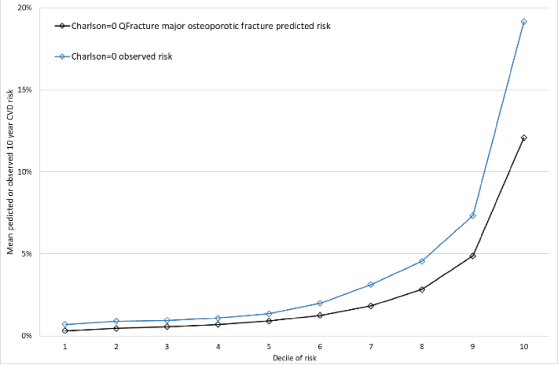

1c: mCCI=1

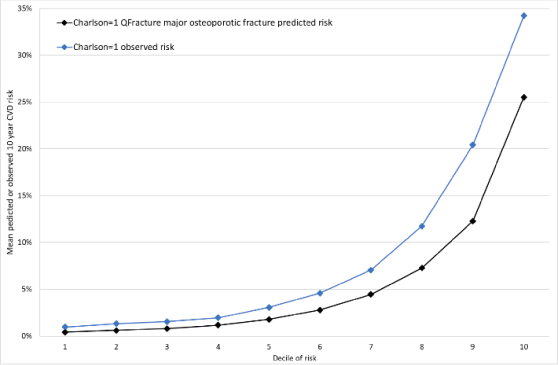

1e: mCCI=2

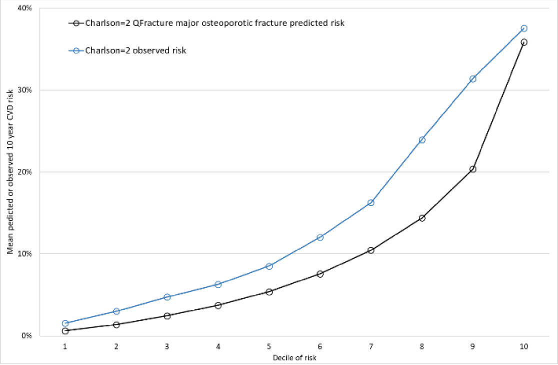

1g: mCCI=3+

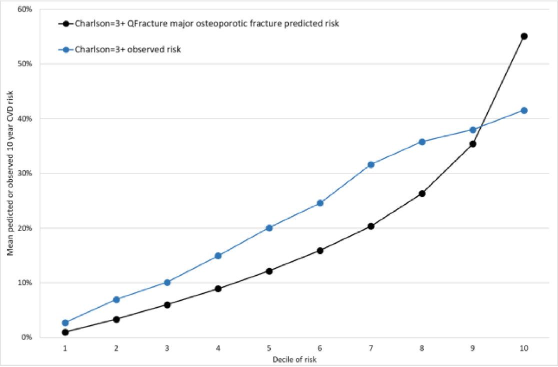

Women (accounting for competing risks)

1b: mCCI=0

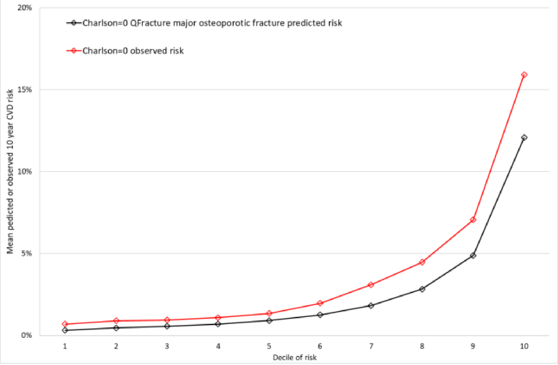

1d: mCCI=1

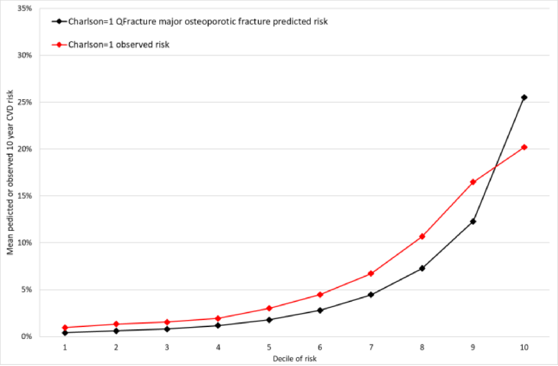

1f: mCCI=2

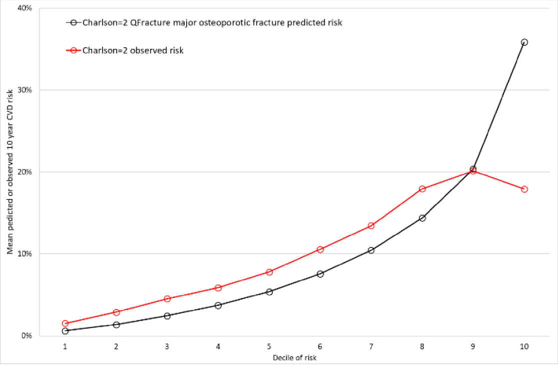

1h: mCCI=3+

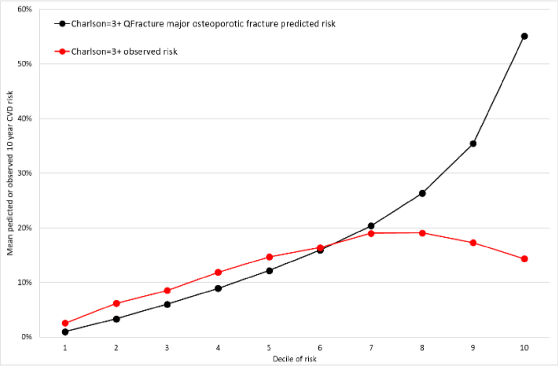

* Observed risk is based on the Kaplan-Meier estimator which does not account for competing mortality risk.
# Observed risk is based on the Aalen-Johansen estimator which accounts for competing mortality risk
Coloured line (observed risk) above matching black line (predicted risk) indicates under-prediction; below indicates over-prediction

Livingstone SJ, *et al. BMJMED* 2022; 1:e000316. doi: 10.1136/bmjmed-2022-000316

Figure S5: Calibration for major osteoporotic fracture in men by Charlson Score without accounting for competing risks (left hand) and accounting for competing risks (right hand)

Men (not accounting for competing risks)

1a: mCCI=0

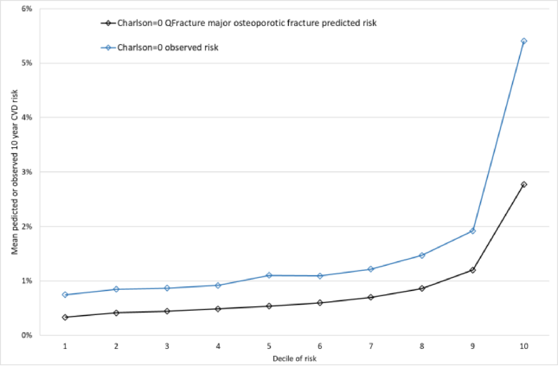

1c: mCCI=1

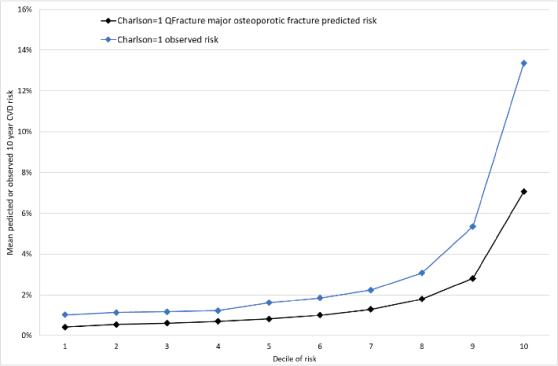

1e: mCCI=2

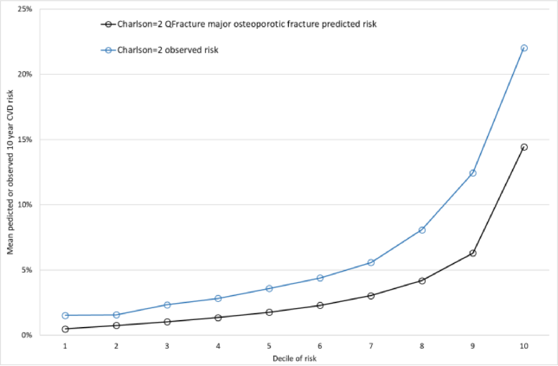

1g: mCCI=3+

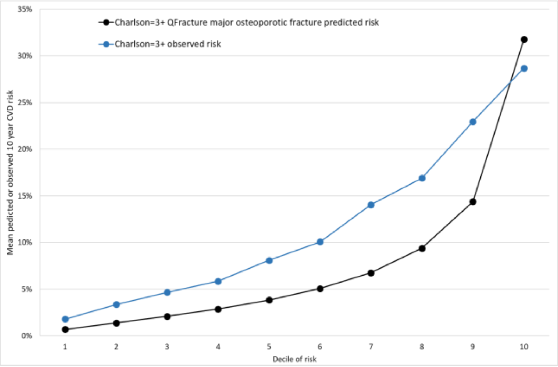

Men (accounting for competing risks)

1b: mCCI=0

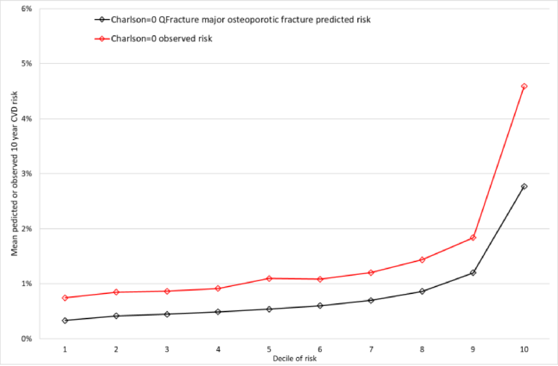

1d: mCCI=1

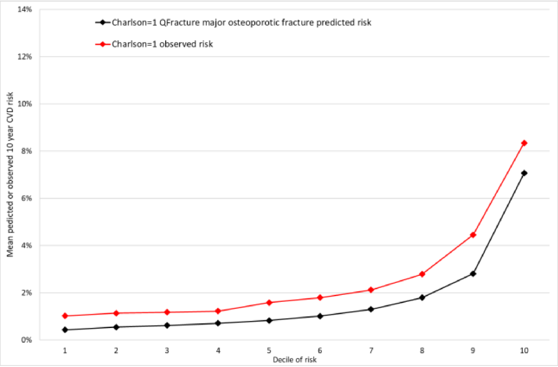

1f: mCCI=2

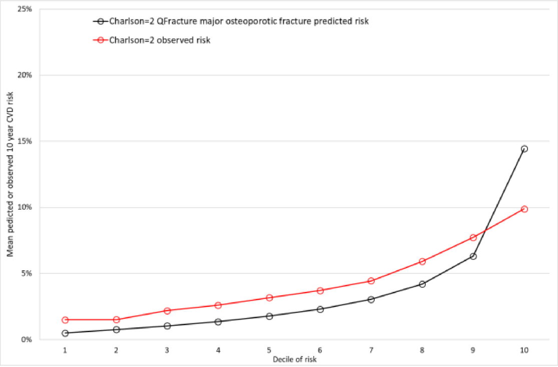

1h: mCCI=3+

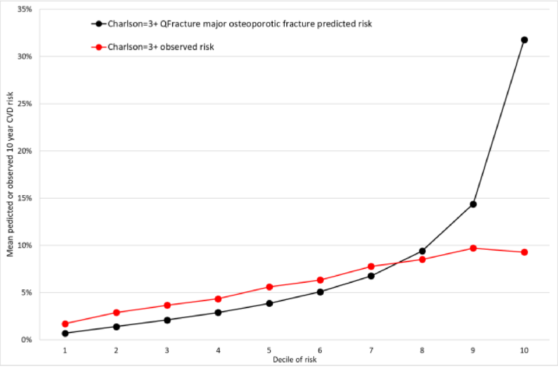

* Observed risk is based on the Kaplan-Meier estimator which does not account for competing mortality risk.
# Observed risk is based on the Aalen-Johansen estimator which accounts for competing mortality risk
Coloured line (observed risk) above matching black line (predicted risk) indicates under-prediction; below indicates over-prediction

Livingstone SJ, *et al. BMJMED* 2022; 1:e000316. doi: 10.1136/bmjmed-2022-000316

Figure S6: Calibration for hip fracture in women by agegroup without accounting for competing risks (left hand) and accounting for competing risks (right hand)

Women (not accounting for competing risks)

1a: Aged 30-64

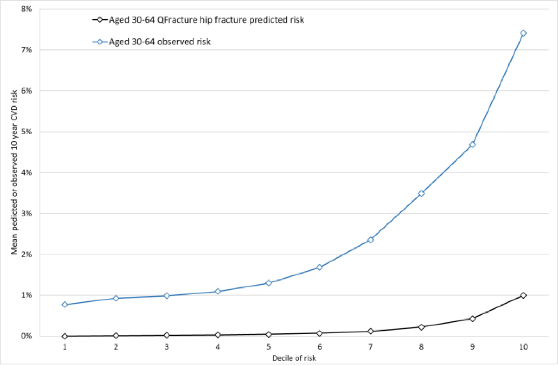

Women (accounting for competing risks)

1b: Aged 30-64

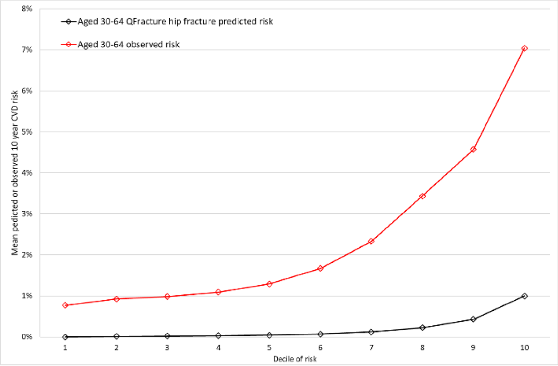

1c: Aged 65-74

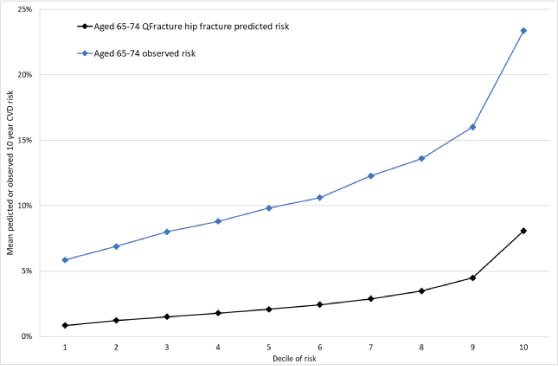

1d: Aged 65-74

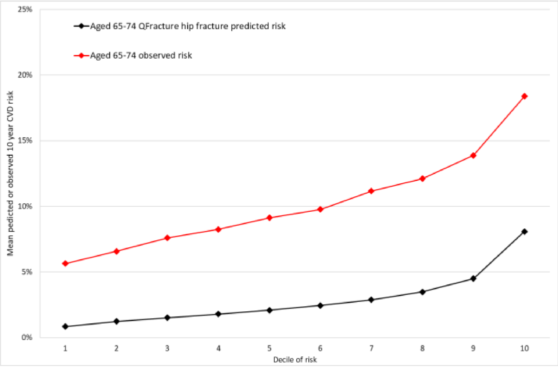

1e: Aged 75-84

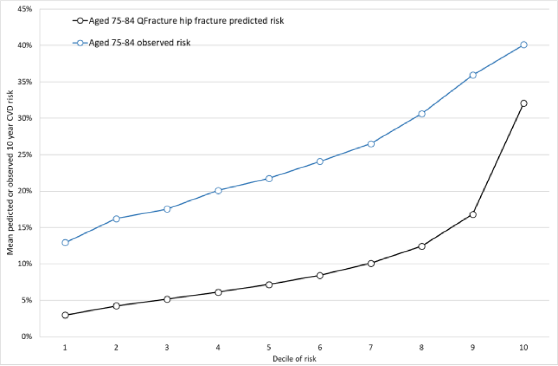

1f: Aged 75-84

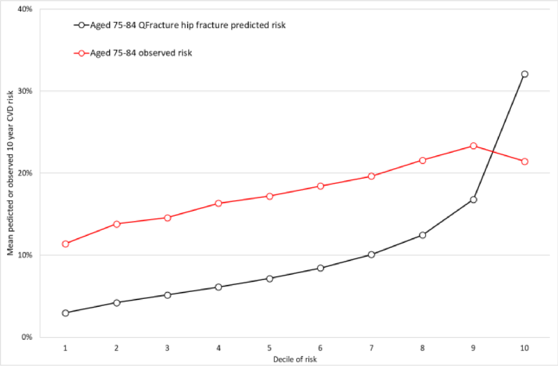

1g: Aged 85-99

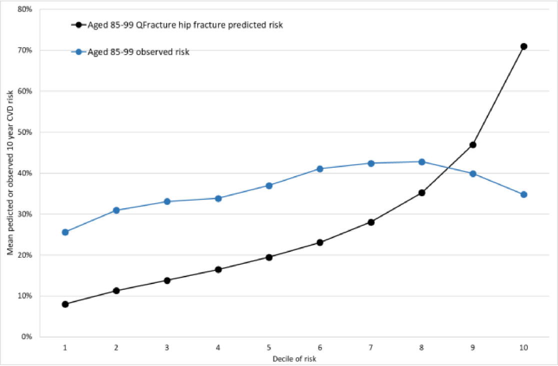

1h: Aged 85-99

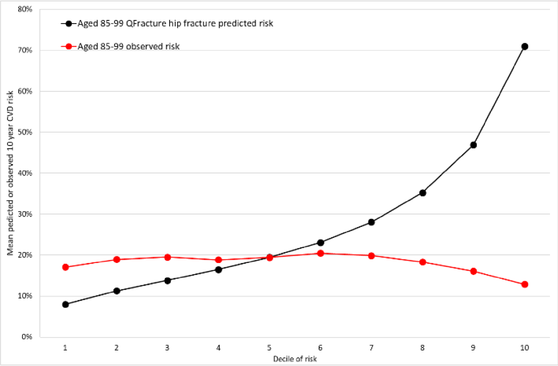

* Observed risk is based on the Kaplan-Meier estimator which does not account for competing mortality risk.
# Observed risk is based on the Aalen-Johansen estimator which accounts for competing mortality risk
Coloured line (observed risk) above matching black line (predicted risk) indicates under-prediction; below indicates over-prediction

Figure S7: Calibration for hip fracture in men by agegroup without accounting for competing risks (left hand) and accounting for competing risks (right hand)

Men (not accounting for competing risks)

1a: Aged 30-64

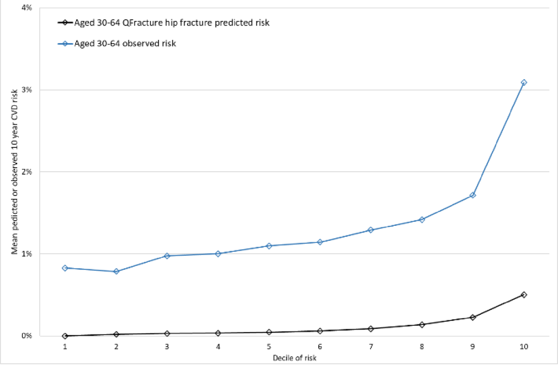

Men (accounting for competing risks)

1b: Aged 30-64

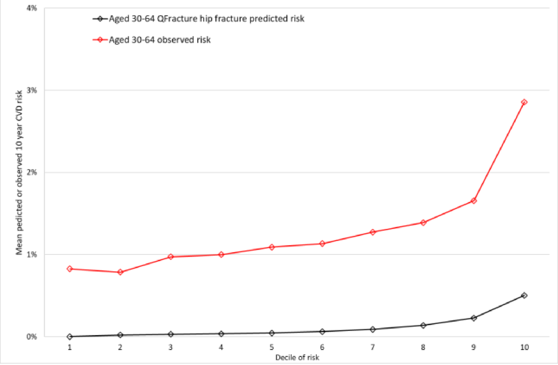

1c: Aged 65-74

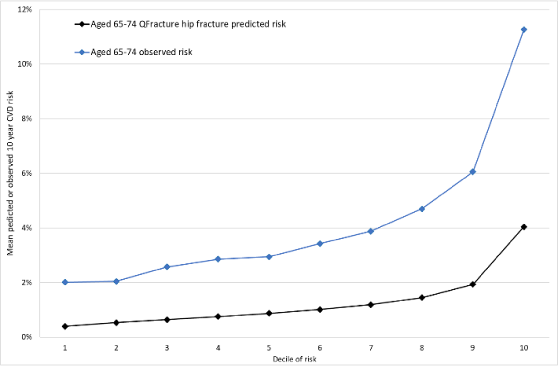

1d: Aged 65-74

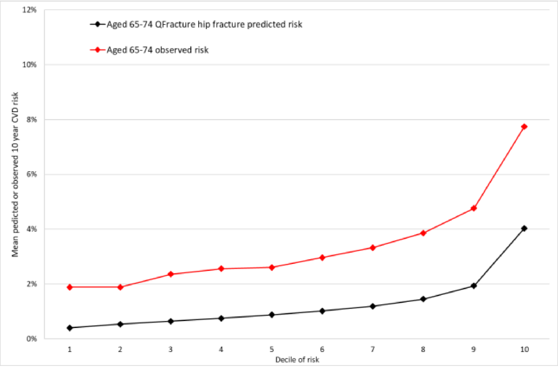

1e: Aged 75-84

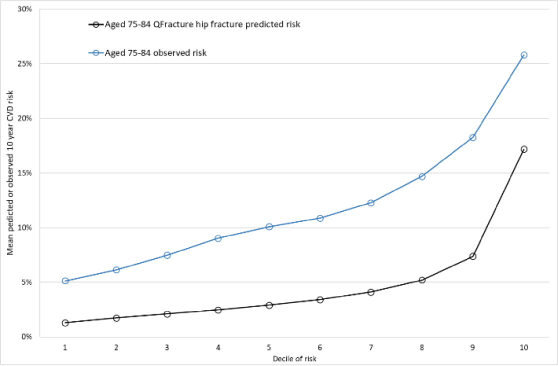

1f: Aged 75-84

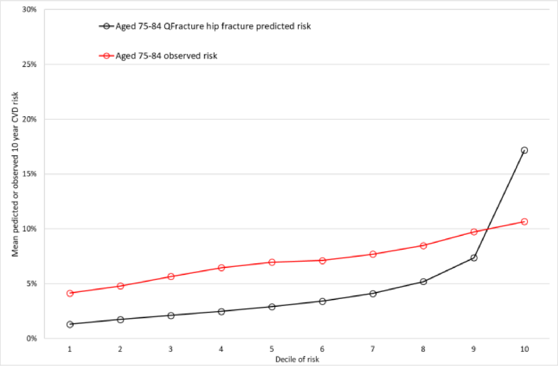

1g: Aged 85-99

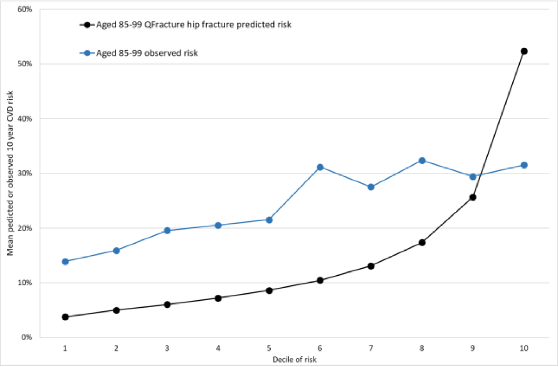

1h: Aged 85-99

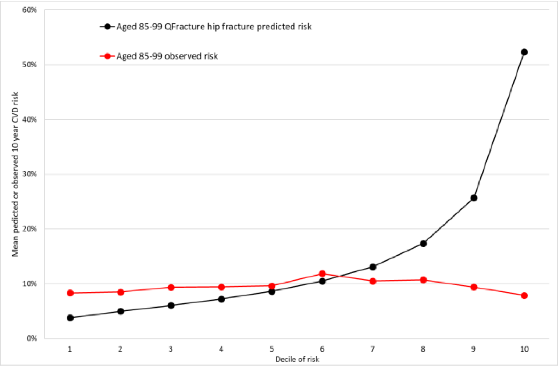

* Observed risk is based on the Kaplan-Meier estimator which does not account for competing mortality risk.
# Observed risk is based on the Aalen-Johansen estimator which accounts for competing mortality risk
Coloured line (observed risk) above matching black line (predicted risk) indicates under-prediction; below indicates over-prediction

Figure S8: Calibration for hip fracture in women by Charlson Score without accounting for competing risks (left hand) and accounting for competing risks (right hand)

Women (not accounting for competing risks)

1a: mCCI=0

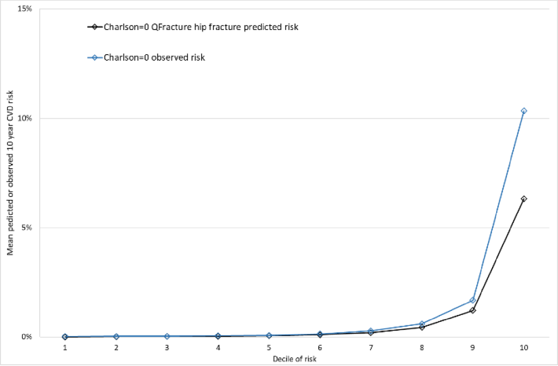

1c: mCCI=1

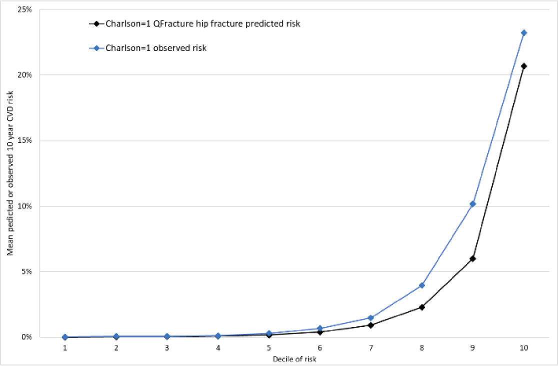

1e: mCCI=2

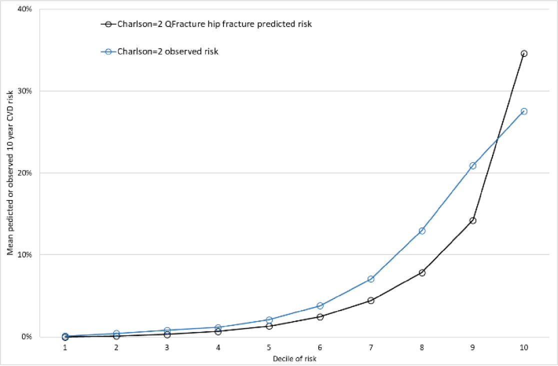

1g: mCCI=3+

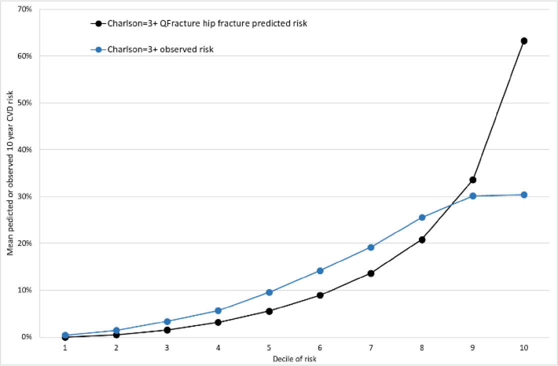

Women (accounting for competing risks)

1b: mCCI=0

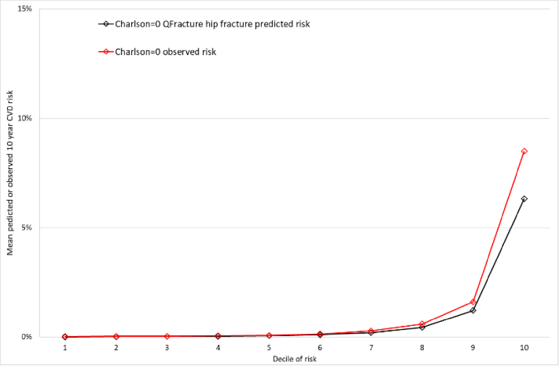

1d: mCCI=1

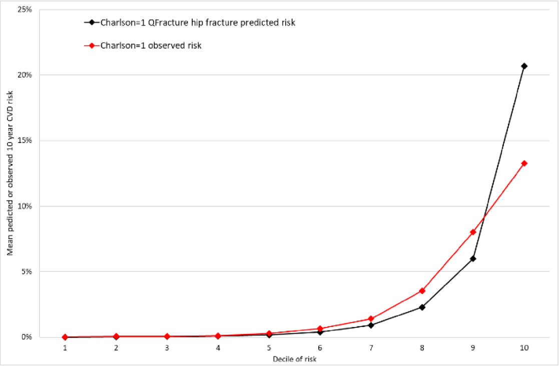

1f: mCCI=2

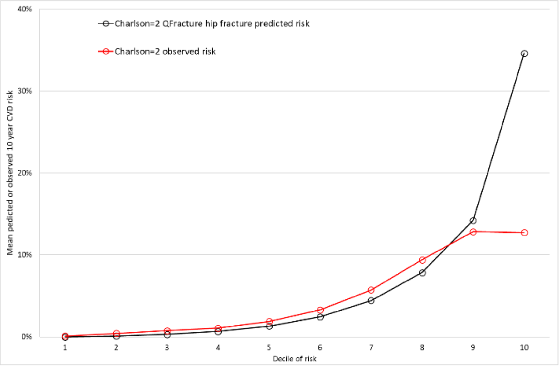

1h: mCCI=3+

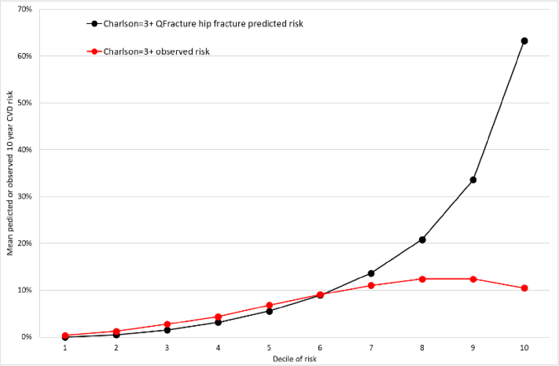

* Observed risk is based on the Kaplan-Meier estimator which does not account for competing mortality risk.
# Observed risk is based on the Aalen-Johansen estimator which accounts for competing mortality risk
Coloured line (observed risk) above matching black line (predicted risk) indicates under-prediction; below indicates over-prediction

Figure S9: Calibration for hip fracture in men by Charlson Score without accounting for competing risks (left hand) and accounting for competing risks (right hand)

Men (not accounting for competing risks)

1a: mCCI=0

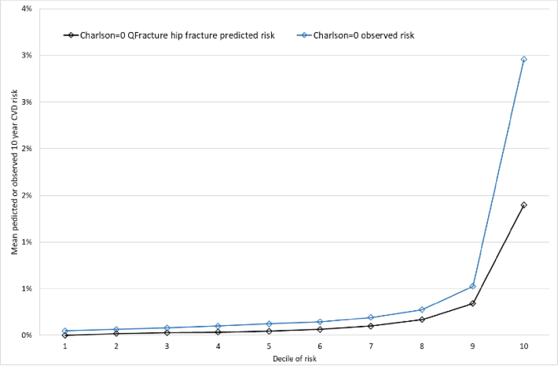

Men (accounting for competing risks)

1b: mCCI=0

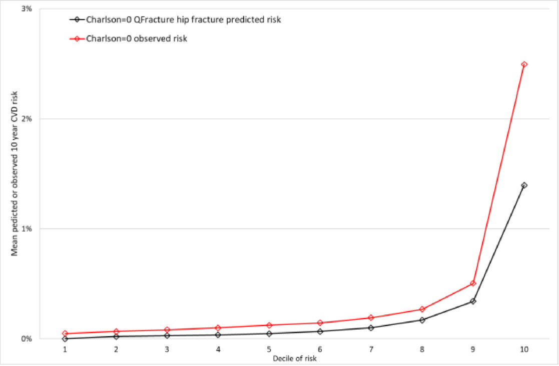

1c: mCCI=1

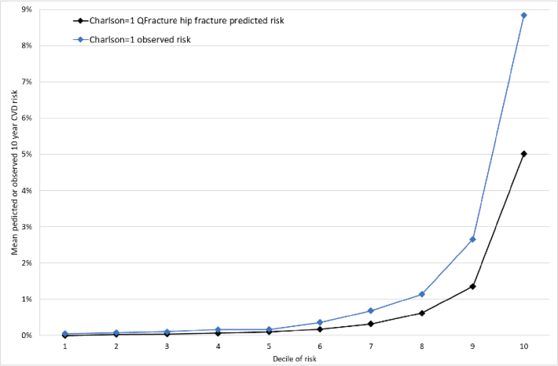

1d: mCCI=1

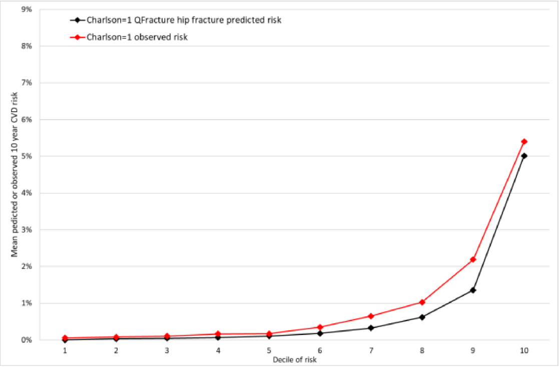

1e: mCCI=2

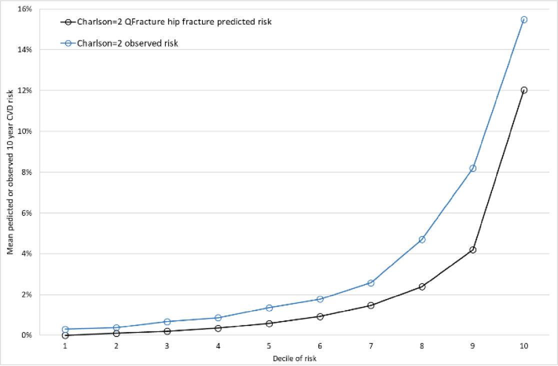

1f: mCCI=2

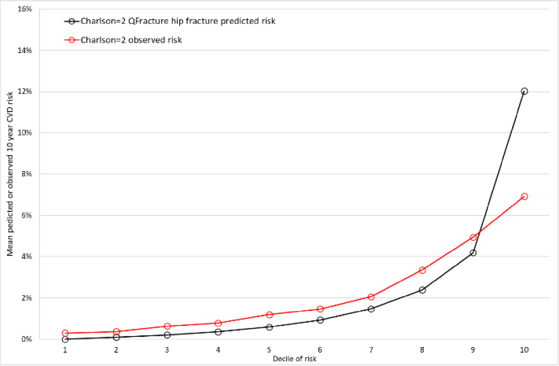

1g: mCCI=3+

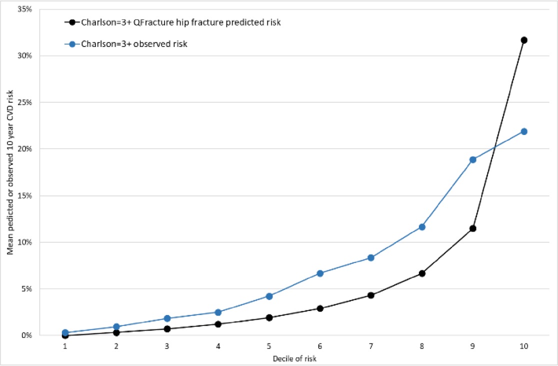

1h: mCCI=3+

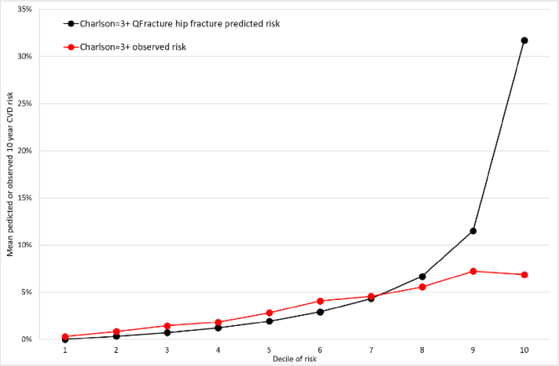

* Observed risk is based on the Kaplan-Meier estimator which does not account for competing mortality risk.
# Observed risk is based on the Aalen-Johansen estimator which accounts for competing mortality risk
Coloured line (observed risk) above matching black line (predicted risk) indicates under-prediction; below indicates over-prediction

References

1. Kuan V, Denaxas S, Gonzalez-Izquierdo A, et al. A chronological map of 308 physical and mental health conditions from 4 million individuals in the English National Health Service. The Lancet Digital Health 2019;1:e63-e77.
2. Livingstone S, Morales DR, Donnan PT, et al. Effect of competing mortality risks on predictive performance of the QRISK3 cardiovascular risk prediction tool in older people and those with comorbidity: external validation population cohort study. The Lancet Healthy Longevity 2021;2:e352-e61.
3. Collins GS, Mallett S, Altman DG. Predicting risk of osteoporotic and hip fracture in the United Kingdom: prospective independent and external validation of QFractureScores2011.
4. Hippisley-Cox J, Coupland C. Predicting risk of osteoporotic fracture in men and women in England and Wales: prospective derivation and validation of QFractureScores2009.
5. Hippisley-Cox J, Coupland C. Derivation and validation of updated QFracture algorithm to predict risk of osteoporotic fracture in primary care in the United Kingdom: prospective open cohort study. BMJ 2012;344:e3427.
